# Permethrin-treated baby wraps for the prevention of malaria: results of a randomized controlled pilot study in rural Uganda

**DOI:** 10.1186/s12936-022-04086-w

**Published:** 2022-02-23

**Authors:** Ross M. Boyce, Enid Muhindo, Emmanuel Baguma, Rabbison Muhindo, Bwambale Shem, Ruthly François, Sam Hawke, Bonnie E. Shook-Sa, Moses Ntaro, Aisha Nalusaji, Dan Nyehangane, Raquel Reyes, Jonathan J. Juliano, Mark J. Siedner, Sarah G. Staedke, Edgar M. Mulogo

**Affiliations:** 1 Institute for Global Health and Infectious Diseases, University of North Carolina at Chapel Hill, 123 West Franklin Street, Suite 230, RM 2151, Chapel Hill, NC 27599 USA; 2 Department of Epidemiology, Gillings School of Global Public Health, University of North Carolina at Chapel Hill, Chapel Hill, NC 27599 USA; 3 Department of Community Health, Faculty of Medicine, Mbarara University of Science and Technology, Mbarara, Uganda; 4 Bugoye Level III Health Center, Uganda Ministry of Health, Kasese, Uganda; 5 Department of Biostatistics, Gillings School of Global Public Health, University of North Carolina at Chapel Hill, Chapel Hill, NC 27599 USA; 6 Epicentre Mbarara Research Centre, Mbarara, Uganda; 7 Department of Medical Laboratory Science, Faculty of Medicine, Mbarara University of Science and Technology, Mbarara, Uganda; 8 Division of Hospital Medicine, UNC School of Medicine, University of North Carolina at Chapel Hill, Chapel Hill, NC 27599 USA; 9 Massachusetts General Hospital and Harvard Medical School, Boston, MA 02114 USA; 10 Department of Clinical Research, London School of Hygiene and Tropical Medicine, London, WC1E 7HT UK

**Keywords:** Malaria, Plasmodium, Permethrin, Insecticide-treated clothing, Prevention

## Abstract

**Background:**

Progress against malaria has stalled and may even be slipping backwards in high-burden countries. This is due to a range of factors including insecticide resistance and mosquito feeding behaviours that limit contact with widely-employed interventions including long-lasting insecticidal nets and indoor-residual spraying. Thus, further innovations in malaria control are urgently needed.

**Methods:**

The pilot was a randomized, placebo-controlled pilot study of permethrin-treated baby wraps—known locally as *lesus*—in children 6–18 months of age at a single site in rural western Uganda. Fifty mother–infant pairs were assigned to permethrin-treated or untreated *lesus* in a 1:1 allocation. Participants and clinical staff were blinded to group assignments through use of sham treatment and re-treatment of *lesus*. Participants attended scheduled clinic visits every 2 weeks for a total 12 weeks. The primary outcome of interest was the safety of the intervention, assessed as changes in the frequency of use, rates of discontinuation, and incidence of adverse events, such as skin rash. Secondary outcomes included acceptability and feasibility of the intervention as measured through participant satisfaction and completion of study activities, respectively.

**Results:**

Overall, rates of retention and participation were relatively high with 86.0% (43 of 50) of participants completing all scheduled visits, including 18 (75.0%) and 25 (96.2%) in the intervention and control arms respectively. By the conclusion of the 12-week follow-up period, one adverse event (0.35 events per 100 person-weeks, one-sided 95% CI 0.0–1.65) was reported. Satisfaction with the *lesu* was high in both groups. In each study arm, there were five incident RDT positive results, but the only PCR-positive results were observed in the control group (n = 2).

**Conclusions:**

Permethrin-treated baby wraps were well-tolerated and broadly acceptable. Adverse events were infrequent and mild. These findings support future trials seeking to determine the efficacy of treated wraps to prevent *P. falciparum* malaria infection in young children as a complementary tool to existing household-based interventions.

*Trial registration*: ClinicalTrials.gov Identifier: NCT04102592, Registered 25 September 2019. Available at: https://clinicaltrials.gov/ct2/show/NCT04102592

**Supplementary Information:**

The online version contains supplementary material available at 10.1186/s12936-022-04086-w.

## Background

Over the past two decades, the burden of *Plasmodium falciparum* malaria has substantially declined with mortality in endemic areas, such as sub-Saharan Africa (SSA) decreasing by more than 35% [1]. The widespread deployment of vector control measures that target indoor-feeding and indoor-resting *Anopheles* mosquitoes, such as long-lasting insecticidal nets (LLIN) and indoor residual spraying (IRS), largely account for these gains [2]. However, these strategies are generally insufficient to interrupt malaria transmission fully [3, 4]. The degree of control that can be attained with LLINs or IRS is limited by a combination of factors including barriers to achieving and sustaining universal LLIN coverage [5–7], the resource-intensive nature of IRS programmes [8, 9], and the emergence of resistance to commonly-employed insecticides [10, 11]. In addition, these household-based interventions can drive selection pressure [12]. For example, LLINs and IRS will favour mosquito behaviours that avoid these interventions, either by feeding on peri-domestic animals, outdoors, or in the early evening when residents are outside the home [13–16].

As evidence of these challenges, reports from the World Health Organization (WHO) suggest that progress against malaria has stalled and may even be slipping backwards in high-burden countries [17]. Interruptions in preventive services, such as LLIN distributions, as a result of the global COVID-19 pandemic may further exacerbate these trends [18]. Even at the peak of progress, however, malaria still accounted for approximately 400,000 deaths per year, with the vast majority occurring among children less than 5 years of age living in rural areas of SSA [17].

Uganda experiences a disproportionate burden of malaria, accounting for 5% of global cases and 3% of global deaths. Malaria is also a common cause of care seeking and healthcare utilization, responsible for approximately 20% of clinical visits [17, 19, 20]. Among endemic countries, Uganda has been a leader in the effort to achieve universal LLIN coverage defined as one net per two household members [21, 22]. Unsurprisingly, Uganda has also observed widespread rise in mosquitoes that are resistant to the first-line insecticides used in LLIN and IRS programmes [11, 23]. Furthermore, there is emerging evidence that the traditional malaria vectors, *Anopheles gambiae* and *Anopheles funestus,* are increasingly exhibiting feeding behaviours that may not bring them into contact with exiting interventions [24], while other vectors such as *Anopheles arabiensis* are playing a larger role in transmission [25]. Thus, further innovations in malaria control are urgently needed [26–30].

**Fig. 1:**
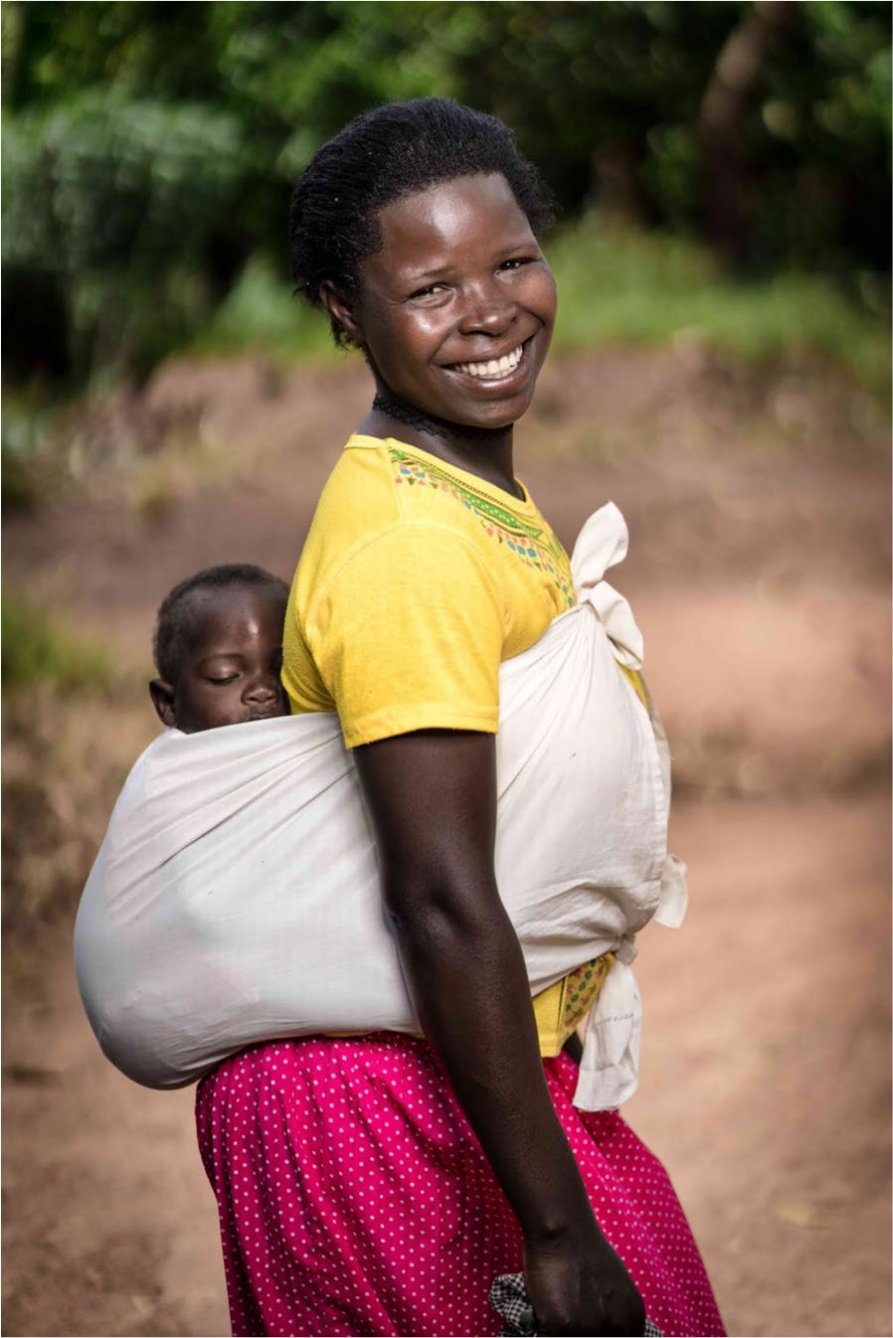
Ugandan woman carrying child in traditional *lesu*. Credit Robert Ditty

In pursuit of this goal, the investigators sought to leverage the traditional practice of mothers carrying young children on their backs utilizing wraps made from locally-purchased cloth as a potential malaria prevention target. The wrap, called a *lesu* in Uganda (Fig. 1), may also serve as a blanket or swaddle for children. Thus, mother and child spend much of the day in contact with the cloth. The stated hypothesis was that when treated with an insecticide or repellent, the *lesu* might provide an additional layer of protection against malaria that would complement existing, household-based interventions. As the first step towards testing this hypothesis, the study team conducted a pilot randomized controlled trial (RCT) assessing the safety, acceptability, and feasibility of using permethrin-treated and untreated *lesus*.

## Methods

### Study site

**Fig. 2:**
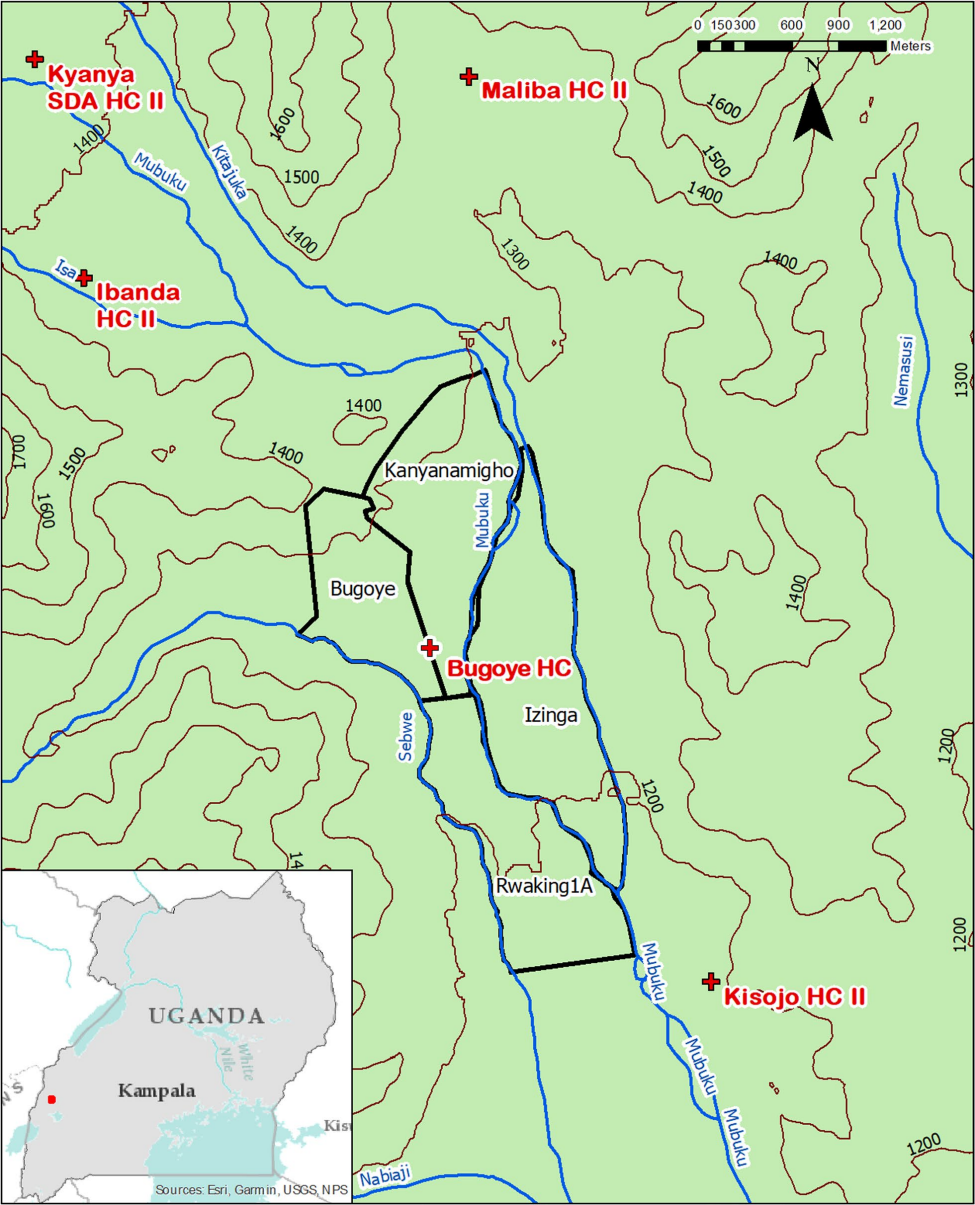
Map of study area in reference to Uganda

The Bugoye Health Center Level III (BHC) is located in the Kasese District of western Uganda (Fig. 2). The topography of the area, situated at the base of the Rwenzori Mountains National Park, is highly varied and characterized by deep river valleys and steep hillsides with elevations up to 2500 m. The climate permits year-round malaria transmission marked by semi-annual transmission peaks typically following the end of the rainy seasons in May and December [31]. The most recent malaria indicator surveys undertaken in the Tooro sub-national region (2018–19) which include the study area, reported *P. falciparum* parasitaemia rates (PfPR) of 7.3% [32]. However, in a recent cross-sectional survey of more than 2,100 households in the Bugoye sub-county, the PfPR among children 2 to 8 years of age was upwards of 30% in many of the low-elevation villages [33]. Similarly, studies from the site have reported that during peak transmission periods malaria may account for 25–35% of pediatric outpatient visits [34, 35].

### Study design

The pilot study was a randomized, placebo-controlled trial of permethrin-treated *lesus* in children 6–18 months of age at enrolment conducted at a single site in rural western Uganda. Fifty mother-infant pairs were assigned in parallel to permethrin-treated or untreated *lesus* in a 1:1 allocation. Participants and clinical staff were blinded to group assignments through use of sham treatment and re-treatment of *lesus*. Participants attended scheduled clinic visits every 2 weeks for a total 12 weeks, after which time participants completed semi-structured interviews about the acceptability of the intervention.

### Intervention

The intervention was provision of *lesus* treated with permethrin to women of children between 6 and 18 months old. Permethrin is a synthetic insecticide with a well-established safety record when used topically to treat scabies and lice [36, 37]. Permethrin has also been widely utilized to treat LLINs and military uniforms for more than a decade [38–41], while treated blankets and tents have been shown to be highly effective in preventing malaria in refugee camps [42, 43]. Permethrin-impregnated clothing has been shown to be effective for preventing mosquito and tick bites among outdoor workers [44, 45]. The U.S. Centers for Disease Control and Prevention (CDC) recommends treatment of clothing with permethrin to prevent mosquito bites in all populations, and specifically reiterated this guidance for pregnant women traveling to Zika-endemic areas to reduce the risk of infection [46]. Notably, permethrin also has a modest repellent effect, which may prevent biting even when mosquitoes are resistant to the killing effect, although results to date are conflicting and primarily derived from studies of *Aedes* mosquitoes [47, 48].

Prior to the study, a series of focus-group discussions were conducted with mothers of young children to inform study protocols, including the target age groups and selection of fabric, size, and style. All *lesus* were procured from a local vendor. Permethrin 40% concentrate was obtained directly from the manufacturer (Sawyer Products, Safety Harbor, FL). Prior to application, permethrin was diluted to 0.5% concentration, a similar concentration used in other applications including military uniforms and outdoor clothing [45, 49]. Intervention *lesus* were soaked in permethrin in accordance with the manufacturer’s instructions. In brief, lesus were rolled tightly, secured with rubber bands, and placed in one gallon plastic bags. Approximately 100 ml of 0.5% permethrin was added, and the bag was gently massaged until the cloth was completely soaked. After one hour, the *lesus* were removed, unrolled, and hung to dry in a shaded area. Control *lesus* underwent similar procedures, but using only water in place of the 0.5% permethrin concentration. Re-treatment of *lesus* with insecticide or placebo occurred every 4 weeks. The retreatment frequency selected was greater than that used with early studies of insecticide-treated nets due to concerns that repeated washing of soiled *lesus* might accelerate permethrin wash-out [50].

### Recruitment and enrolment

Sensitization meetings were conducted with teams of community health workers (CHW) from the four villages closest to BHC. During the session, staff described the study objectives, general methods, and eligibility criteria (Table 1). The CHWs, each of whom is responsible for approximately 30 households, communicated information about the study to women with children 6 to 18 months of age in their respective coverage areas. Women expressing interest provided their names and contact information. After canvassing was complete, staff coordinated four small-group meetings each consisting of 15–20 interested women. At each meeting, study staff provided detailed information about the study, including objectives, methods, risks, and benefits in the local language (e.g., Lhukonjo). Attendees were offered opportunities to ask questions both in the group setting and later in a private area. Women agreeing to participate in the study then provided written consent.

**Table 1:** Eligibility criteria for study participation

	Criteria	Rationale
1	Mother ≥ 18 years of age	Age required to provide written informed consent
2	Child 6–18 months of age	Lesu use declines after 24 months of age
3	Resident of one of four villages in the subcounty	Villages with relatively high malaria transmission and in close proximity to the health center
4	HIV negative	Women living with HIV and HIV-exposed children likely to be taking cotrimoxazole prophylaxis, which may impact risk of malaria
5	Willing to provide written informed consent and adhere to protocols	Required for all studies in accordance with applicable guidelines and regulations governing research

### Household visit

Initial home visits were conducted upon enrolment to document demographic and household characteristics, including malaria knowledge and care-seeking behaviours using a modified questionnaire from the most recent Uganda Demographic and Health Survey [51] (Additional file 1). As cotrimoxazole preventive therapy (CPT), which is highly effective in preventing malaria, is considered standard-of-care for all people living with HIV as well as HIV-exposed infants, we performed testing using a rapid, point-of-care HIV 1/2 test (SD Bioline HIV-1/2 3.0) prior to finalizing enrolment [52, 53]. Given concerns about testing in a more public setting conveyed during the focus groups, screening for HIV was performed during the household visit. Upon completion of the survey, all participants received a new LLIN (Permanet 2.0, Vestergaard S.A., Switzerland) with guidance that the net was intended for the participating child.

### Randomization

The randomization sequence was generated using the *runiform* function in Stata (StataCorp LLC. College Station, USA), with a 1:1 allocation between permethrin-treated (intervention) or sham-treated (control) arms. Only the principal investigator and study coordinator had access to the allocation plan. Prior to the baseline clinic visit, participating mother-infant pairs were assigned a unique study number with a corresponding treatment assignment.

### Baseline visit

On the scheduled date, participants presented to the BHC study clinic where a Clinical Officer (CO) obtained a focused health history (Additional file 1) and performed a physical examination, including measurement of axillary temperature, height, weight, and mid-upper-arm circumference (MUAC) as well as a full examination of the skin. Capillary blood was collected to test for malaria using the histidine rich protein-2 (HRP2)-based rapid diagnostic test (SD Bioline Malaria Ag P.f, Abbott Laboratories, USA) and quantification of haemoglobin (Hb) with a point-of-care analyzer (Hemocue Hb 201 + , Hemocue America, USA). Approximately 50 µL of blood was aliquoted from the blood collection tube and placed onto filter paper (Whatman 903, Chicago, USA) to make five dried blood spots (DBS) for polymerase chain reaction (PCR) testing and long-term storage. At the conclusion of the enrolment visit, participants received one treated- or untreated-*lesu* for routine use and instructed to use the study *lesu* exclusively.

### Follow-up visits

Evaluations and activities specific to each visit are listed in Table 2. Every 2 weeks, participants attended study visits where clinical staff assessed mothers and infants for adverse reactions, performed testing for malaria using an RDT, and collected capillary blood for DBS preparation. Research staff administered a brief questionnaire of recent medical history (e.g., rash, fever), care-seeking behaviour, and frequency of *lesu* use and washing (Additional file 1).

**Table 2:** Schedule of data collection activities to include questionnaires and laboratory testing

	Study encounter							
	Home visit	Baseline	Week 2	Week 4	Week 6	Week 8	Week 10	Week 12
**Mother**								
Baseline survey	X							
HIV-1/2 RDT	X							
Venous blood		X						X
Haemoglobin		X						X
Exit interview								X
**Child**								
Health update		X	X	X	X	X	X	X
Exam		X	X	X	X	X	X	X
Height/weight		X						X
MUAC		X						X
Venous blood		X						X
Haemoglobin		X						X
Malaria RDT		X						X
DBS		X						X
Capillary blood			X	X	X	X	X	
Malaria RDT			X	X	X	X	X	
DBS			X	X	X	X	X	
**Lesu**								
Treatment		X						
Re-treatment				X		X		

Participants who were lost to follow-up, defined as a participant who missed an appointment and could not be contacted or located by study staff after 1 week, were replaced with another eligible individual if the loss occurred before the Week 6 visit. Participants who were lost to follow-up after the Week 6 visit were not replaced.

### Laboratory testing

Molecular diagnosis of malaria was completed at the Epicentre Mbarara Research Centre. DNA from DBS was extracted using commercial extraction kits (Quick-DNA Miniprep, Zymo Research, USA) in accordance with the manufacturer’s instructions. After extraction was complete, we used a high-resolution melt (HRM) master mix (Qiagen, Hilden, Germany) and primers targeting the 18S rRNA genes as previously described [54]. Thermocycling, fluorescent detection, and HRM steps were performed in a Rotor-Gene Q real-time PCR instrument, using a 72-well rotor (Qiagen).

### Statistical analysis

Study data were recorded in REDCap, a secure electronic database using tablet devices equipped with internet connectivity [55]. The primary outcome of interest was the safety of the intervention, assessed as the frequency of adverse events such as skin rash, lesion, or any other symptom suspicious for cutaneous irritation or inflammation. The rate of discontinuation of *lesu* use among participating mother–child dyads was also recorded. Secondary outcomes included acceptability and feasibility of the intervention as measured through participant satisfaction and completion of study activities, respectively. Additional exploratory outcomes included: (i) the incidence of clinical malaria requiring treatment, (ii) the incidence of RDT positivity (iii) the change in Hb concentration from baseline to the final (e.g., 12-week) visit, and (iv) change in MUAC from baseline to the final visit. Due to the pilot nature of the study, no power calculations were conducted for the primary or secondary outcomes [56, 57].

Data were analysed according to the treatment participants received (i.e., modified intention-to-treat). Descriptive statistics were computed for all outcomes of interest stratified by arm, with sample sizes and proportions presented for categorical variables and medians and interquartile ranges (IQR) for continuous variables. Incidence rates for self-reported, recurrent events including fever, care seeking, and malaria treatment were estimated as events per time at risk and are shown as events per 100 person weeks with corresponding exact 95% confidence intervals (CIs). Given the pilot nature of the study with no planned statistical testing, small sample sizes, and resulting lack of statistical power to detect differences in outcomes between arms, no formal statistical tests of significance were performed. The rank-based Hodges-Lehmann estimator was used to estimate differences in weight, haemoglobin, and MUAC between arms with corresponding exact 95% CIs. All statistical analyses were conducted using R version 4.0.5.

### Ethical approvals

Study procedures were approved by the University of North Carolina Institutional Review Board (18–1819), the Mbarara University of Science and Technology Research Ethics Committee (05/08–18), and the Uganda National Council of Science and Technology (SS 4833).

## Results

**Fig. 3:**
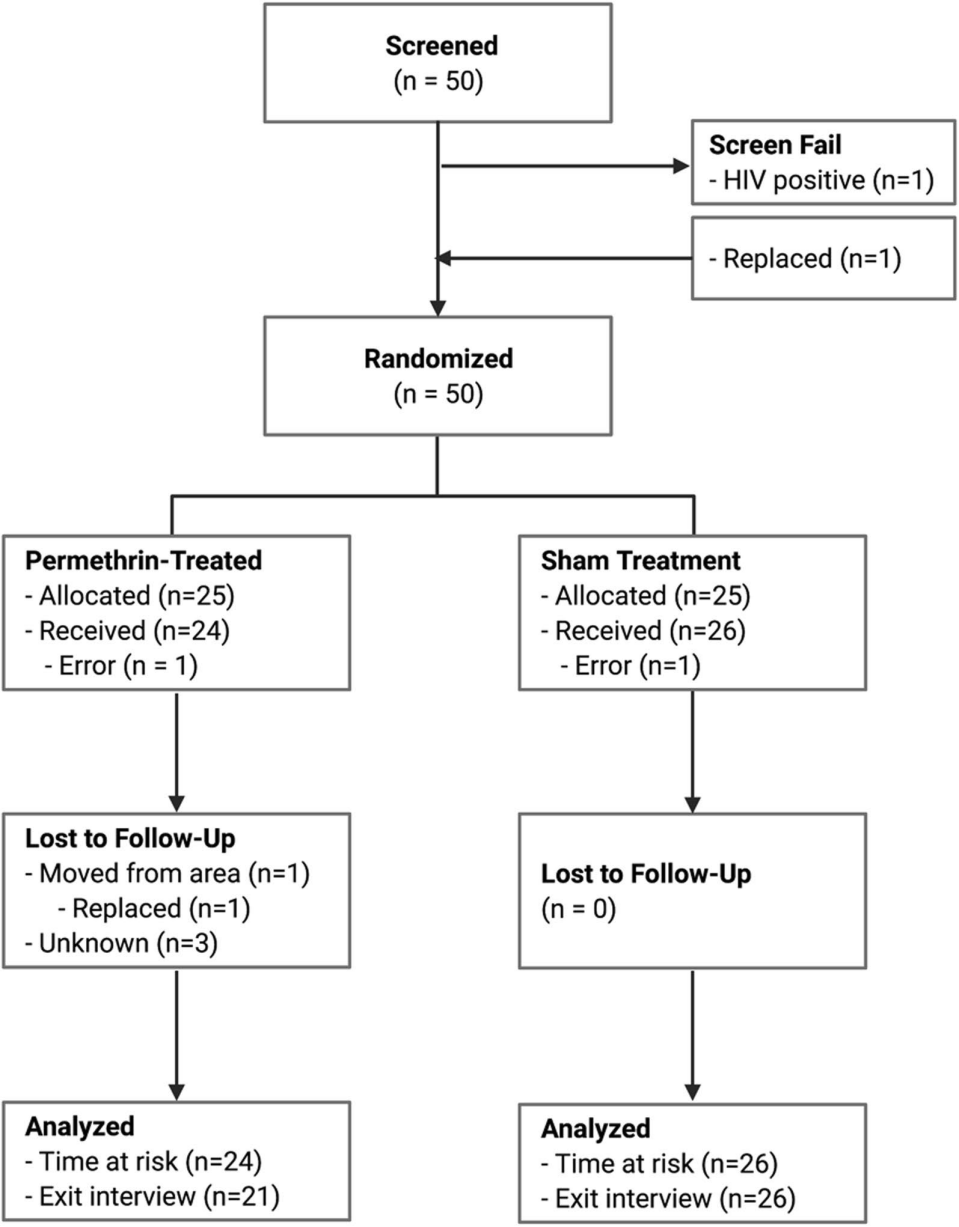
CONSORT Flow Diagram

After screening and eligibility assessment, 50 women-child pairs were enrolled in the study. Baseline household visits were conducted in November 2019. One participant tested positive for HIV during the household visit. This participant was immediately linked to care, but was excluded from further participation and replaced by another participant (Fig. 3). Randomization and baseline visits took place approximately 2 weeks after the household visits.

Characteristics of participating mother-infant pairs are summarized in Table 3. Overall, caregivers had relatively low educational attainment, multiple children in the home, and limited resources, although these characteristics were generally balanced between the arms. On the exit interview, all mothers reported using the *lesu* to carry the child on their back. Additionally, about one-third (n = 17, 34%) also reported using the *lesu* as a blanket to put the child down to sleep (Table 4) with a higher proportion of mothers in the intervention group (52.4% vs 23.1%) using the *lesu* for this purpose compared to the control group. Mothers in both groups reported washing *lesus* a median of approximately four times every per 2-week period.

**Table 3:** Baseline characteristics of study participants including both mothers and children

	Control group	Intervention group
	N = 26	N = 24
**Mothers**		
Age (median, IQR)	26 (23–31)	24 (21–26.3)
Highest level of school (n, %)		
Primary	14 (58.3)	15 (62.5)
Secondary	10 (41.7)	6 (25.0)
High School or University	0 (0.0)	3 (12.5)
Reading ability (n, %)		
Unable to read card	6 (24.0)	2 (8.7)
Able to read parts of sentence	9 (36.0)	13 (60.9)
Able to read whole sentence	10 (40.0)	7 (30.4)
Number of births (median, IQR)	4 (1–5)	3 (1–4)
Took IPTp during this pregnancy	25 (96.2)	24 (100)
Number of doses of IPTp (median, IQR)	3 (2.75–4)	3 (3–4)
Ever hospitalized (n, %)	8 (32.0)	6 (26.1)
Take any medication every day (n, %)	0 (0)	1 (4.2)
Hb level (g/dL)	13.0 (12.1–14.2)	12.9 (12.5–13.7)
**Children**		
Weight (kg)	9.0 (7.9–9.5)	8.4 (8.0–9.3)
MUAC (cm)	15.2 (14.2–16)	14.4 (13.9–15.0)
Hb level (g/dL)	10.1 (9.5–10.8)	10.3 (9.9–10.8)
Slept under net last night (n, %)	25 (96.2)	21 (87.5)
Fever in last 2 weeks (n, %)	10 (38.5)	12 (50.0)
**Household (n, %)**		
Earthen or sand floor?	14 (53.8)	14 (58.3)
Electricity in the house?	6 (24.0)	4 (18.2)
Source of water in the house or yard?	9 (34.6)	6 (25.0)
Own a mobile phone?	23 (92.0)	18 (85.7)
Own any livestock?	17 (68.0)	16 (72.7)
Have a bank account?	15 (60.0)	9 (40.9)
Number of LLINs in home (median, IQR)	1 (1–2)	2 (1–2)

*Hb*  haemoglobin, *IQR*  interquartile range, *LLIN*  long-lasting insecticidal net, *MUAC*  mid-upper arm circumference

**Table 4:** Summary of reported *lesu* use and outcomes of interest

	**Control group**	**Intervention group**
	N = 26	N = 24
*Safety outcomes*		
Adverse events (n, %)		
Child	0 (0)	1 (4.2)
Mother	0 (0)	0 (0)
*Feasibility*		
Attendance (n, %)		
Median number of follow-up visits (IQR)	6 (6–6)	6 (5–6)
Completed both re-treatments	25 (96.2)	21 (87.5)
Completed exit Interview	26 (100.0)	21 (87.5)
Completed all visits	25 (96.2)	18 (75.0)
*Acceptability and use*		
Daily use of *lesu *(n, %)		
Carry child on back	26 (100.0)	21 (100.0)*
Blanket/swaddle for sleep	6 (23.1)	11 (52.4)*
Blanket for sitting	0 (0)	0 (0)
Washing (median, IQR)		
Reported washes per 2-week period	4.0 (2.3–6.0)	4.0 (3.0–6.0)
Satisfaction (n, %)		
Would recommend *lesu* to others	26 (100.0)	21 (100.0)*
Would be willing to pay more for *lesu*	26 (100.0)	21 (100.0)*
*Exploratory outcomes*		
Illness (events per 100 person-weeks, 95% CI)		
Fever	7.0 (4.2–11.1)	8.1 (5.0–12.4)
Care seeking for any cause	17.0 (12.5–22.7)	16.8 (12.1–22.7)
Malaria treatment received	3.0 (1.3–5.8)	6.0 (3.4–9.9)
Malaria testing		
Incident RDT positives (n)	5	5
RDT positive prevalence (%)	10.7	8.4
PCR Positive (n)	2	0 (0)
Change growth measures^a^ (median, IQR)		
Change in weight (kg)	0.9 (0.5–1.3)	0.6 (0.4–0.9)
Change in MUAC (cm)	− 0.85 (− 1.2–0.3)	0.2 (− 0.2–0.7)
Change in Hb (g/dL)	0.88 (0.5–1.3)	0.78 (0.3–1.2)

*Hb* haemoglobin, *IQR*  interquartile range, *MUAC*  mid-upper arm circumference, *RDT*  rapid diagnostic test

^a^Change computed as 12-week measure minus baseline measure

*Three participants lost to follow-up did not complete exit interview

Rates of retention and participation were relatively high with 86.0% (43 of 50) of participants completing all scheduled visits, including 18 (75.0%) and 25 (96.2%) in the intervention and control arms respectively. Similarly, 24 (100%) of participants in the intervention and 26 (100%) in the control arm returned for re-treatment visit. Overall, 94% (47 of 50) of participants completing the final visit and exit interview, when accounting for replacements.

By the conclusion of the 12-week follow-up period, one adverse event (0.35 events per 100 person-weeks, one-sided 95% CI 0.0–1.65)—a transient, pruritic rash—was reported in a child in the intervention group. This rash had resolved before a scheduled visit and was not able to be confirmed at the time of physical examination. The event did not prompt discontinuation of the *lesu*. No side effects of *lesu* use were reported by mothers in either arm. Satisfaction with the *lesu* was high in both groups, with all responding participants willing to recommend the *lesu* and pay extra for a treated *lesu*.

Reports of clinical illness, including subjective fever, care-seeking for any cause, and administration of an antimalarial drug in the 2-week period between visits were generally similar between groups (Table 4). In each study arm, there were five incident RDT positive results, defined as a negative test result during the previous visit followed by a positive test result. The only PCR-positive results, indicative of *P. falciparum* parasitemia at the time of specimen collection, were observed in the control group, both of whom had corresponding RDT positive results. Observed changes in haemoglobin level from enrolment to the final visit was 0.78 g/dL (95% CI 0.3–1.2) higher in the intervention and 0.88 g/dL (95% CI 0.5–1.3) higher in the control group from baseline to study completion, while changes in MUAC were 0.2 cm higher (95% CI − 0.2–0.7) and − 0.85 cm (− 1.2–0.3) lower, respectively (∆ = 0.7 cm, 95% CI 0.2 to 1.3).

## Discussion

This pilot study demonstrates that permethrin-treated baby wraps were well-tolerated, feasible and acceptable to women with young children in Uganda. Only one adverse event out of 24 women-child pairs in the permethrin-treated group was detected, which was described as mild and resolved without treatment or discontinuation of the intervention. These findings are consistent with previous studies of permethrin-treated clothing and materials and provide further evidence to support a larger trial to evaluate the efficacy of the intervention to prevent malaria infection. This approach has many potential advantages over current standard of care malaria control interventions, including: (i) targeting the most vulnerable (i.e., young children), (ii) integrating with existing cultural norms, and (iii) complementing current prevention strategies by offering protection against outdoor- and/or day-time biting *Anopheles* mosquitoes. Such an intervention could also be valuable in situations where LLINs and IRS may not be practical, including among nomadic populations and amidst humanitarian emergencies.

While not designed or adequately powered to measure efficacy, the study did identify some preliminary results of interest. Perhaps most promising is that the only two PCR-confirmed infections observed during the study period occurred in children in the control group. With the small number of events, however, it is possible, if not likely, that this finding was attributable to the timing of sample collection with the two positive results occurring in individuals who had been infected shortly before the clinic visit. In contrast, other participants may have sought care and received treatment prior to the visit, which would be expected to clear the parasitemia, although the RDT may have remained positive due to persistent HRP2 antigenaemia [58]. Notably, reported rates of care seeking and malaria treatment were similar in the two arms, which would be consistent with this explanation. Future trials will require active surveillance strategies to accurately test for differences in the incidence of clinical disease.

The modest increase in MUAC among children in the intervention arm also requires further exploration. While prevention of malaria may underlie this finding, it is also possible that reductions in contact with blood feeding insects (e.g., bedbugs) [59] may also be relevant. The study did not assess for these outcomes, but this may be another benefit of permethrin treatment. However, the lack of pre-specified design and small number of participants substantially also limits the ability to interpret these results. Furthermore, the absence of correlation with changes in weight or Hb concentration also dampers our ability to assert positive conclusions.

Lastly, all three children who were lost to follow-up had been randomized to the intervention group. While it is most likely that this situation occurred by chance, it cannot excluded that these individuals discontinued participation due to side effects or low acceptability of the permethrin-treated *lesu*. If this were true, the findings would underestimate the rate of adverse events and overstate the tolerability and/or acceptability of the intervention.

This study has a number of strengths, including the randomization of participants, use of sham-treatment with blinding of participants and clinic staff, and design of the intervention, which is based on existing cultural childcare practices. The study also has important limitations, foremost of which is the lack of appropriate surveillance methods and statistical power to detect efficacy. A larger clinical trial powered to detect clinically significant effects of the *lesus* on malaria incidence is planned. Second, there was no entomological surveillance conducted in parallel to clinical outcomes. Therefore, the study does not directly estimate the potential effect of the intervention on mosquito landing and feeding. Assuming the sporozoite rate is relatively low and most mosquitoes are not harbouring parasites, it is possible that treated *lesus* could reduce mosquito-human contact, while not showing substantial differences in the incidence of malaria.

To explore this further, paired baseline and end visit samples for *Anopheles* mosquito salivary antigen (e.g., gSG6-P1) will be tested [60]. Study staff did not directly observe each participant’s frequency and duration of *lesu* use or measure the level of permethrin in individual lesus prior to re-treatment. Unmeasured differences between arms could impact the interpretation of the findings and should be incorporated into future studies. Despite these limitations, the study did achieve its primary aim, namely assessing the tolerability and acceptability of the intervention, while also successfully piloting protocols and data collection methods in support of future studies.

## Conclusions

Permethrin-treated baby wraps were well-tolerated and broadly acceptable. Adverse events were infrequent and mild. Results in regard to child health and specifically malaria infection are intriguing, but require further study. These findings support future trials seeking to determine the efficacy of treated wraps to prevent *P. falciparum* malaria infection in young children as a complementary tool to existing household-based interventions.

## Supplementary Information

**Additional file 1.** Baseline Survey.

## Data Availability

Deidentified individual data that supports the results will be shared beginning 9 to 36 months following publication provided the investigator who proposes to use the data has approval from an Institutional Review Board (IRB), Independent Ethics Committee (IEC), or Research Ethics Board (REB), as applicable, and executes a data use/sharing agreement with UNC.
