# Supplementary material for: Permethrin-treated baby wraps for the prevention of malaria: results of a randomized controlled pilot study in rural Uganda
Source: Malar J. 2022 Feb 23;21:63. doi: 10.1186/s12936-022-04086-w (PMC8864600; doi:10.1186/s12936-022-04086-w)
Supplement: Supplementary file 1 — Additional file 1. Baseline Survey. [file 12936_2022_4086_MOESM1_ESM.pdf]

REDCap Global - International Studies @ UNC

UNC - Chapel Hill
TraCS Institute

Boyce - CFH Foundation PID 150

Project Home | Project Setup | Online Designer | Data Dictionary | Codebook

Data Dictionary Codebook

02/05/2021 11:26am

Collapse all instruments

	#	Variable / Field Name	Field Label<br>*Field Note*	Field Attributes (Field Type, Validation, Choices, Calculations, etc.)
		Instrument: **Initial Visit** (initial_visit)		Collapse
	1	study_id	Study ID	text
	2	unique	Unique Identifier<br>*MUST-UNC Lesu / ###*	text (integer, Min: 000, Max: 055)
	3	date_visit_v0	Section Header: *Initial Visit*<br>Date of visit<br>*DD-MM-YYYY*	text (date_dmy), Required
	4	height_v0	Section Header: *I. Vital Signs - Child*<br>Height<br>*cm*	text (number, Min: 50, Max: 100), Required
	5	weight_v0	Weight<br>*kg*	text (number, Min: 5, Max: 20), Required
	6	muac_v0	Mid-Upper Arm Circumference<br>*cm*	text (number, Min: 5, Max: 25), Required
	7	temp_v0	Axillary temperature<br>*degrees Celsius*	text (number, Min: 35, Max: 45), Required
	8	bednet_v0	Section Header: *II. Medical History - Child*<br>Did the child sleep under a bed net last night?	yesno, Required<br>1 Yes<br>0 No
	9	fever_v0	Has the child had fever in last two weeks?	yesno, Required<br>1 Yes<br>0 No
	10	onset_v0<br>Show the field ONLY if:<br>[fever_v0] = '1'	If yes, when did the fever start<br>*DD-MM-YYYY*	text (date_dmy)
	11	sick_v0<br>Show the field ONLY if:<br>[fever_v0] = '0'	Even if the child has not had a fever, has he or she been otherwise unwell?	yesno, Required<br>1 Yes<br>0 No
	12	symp_v0<br>Show the field ONLY if:<br>[sick_v0] = '1'	If yes, what symptoms has the child experienced?	checkbox<br>0 symp_v0___0 Cough<br>1 symp_v0___1 Diarrhea<br>2 symp_v0___2 Ear Ache

				3 symp_v0___3 Not feeding<br>4 symp_v0___4 Rash<br>5 symp_v0___5 Runny nose<br>6 symp_v0___6 Other (specify below)
	13	symp_other_v0<br>Show the field ONLY if:<br>[symp_v0(6)] = '1'	List other symptoms:	notes
	14	healthcentre_v0<br>Show the field ONLY if:<br>[fever_v0] = '1' or [sick_v0] = '1<br>'	Has the child been seen at a hospital, health centre, clinic, drug shop, or other medical attendant for these symptoms?	yesno<br>1 Yes<br>0 No
	15	hc_where_v0<br>Show the field ONLY if:<br>[healthcentre_v0] = '1'	If yes, where?	radio<br>0 Hospital<br>1 Health Centre<br>2 Drug Shop or Pharmacy<br>3 VHT<br>4 Traditional Medicine
	16	medicine_v0<br>Show the field ONLY if:<br>[healthcentre_v0] = '1'	Did the child receive medicine for malaria?	yesno<br>1 Yes<br>0 No
	17	med_date_v0<br>Show the field ONLY if:<br>[medicine_v0] = '1'	When did the child take the last dose (i.e. pill) of medicine?<br>*DD-MM-YYYY*	text (date_dmy)
	18	mrdt_v0	Section Header: *III. Laboratory Testing - Child*<br>Malaria RDT performed?	yesno, Required<br>1 Yes<br>0 No
	19	mrdt_res_v0<br>Show the field ONLY if:<br>[mrdt_v0] = '1'	Malaria RDT Result<br>*Repeat any invalid tests*	radio<br>0 Negative<br>1 Positive
	20	treat_v0<br>Show the field ONLY if:<br>([temp_v0] >= 37.5 or [fever_v<br>0] = '1') and [mrdt_res_v0] = '1'	If mother reported fever or child's temperature was >37.5 C, which antimalarial treatment provided?	radio<br>0 None (explain below)<br>1 Coartem<br>2 Quinine<br>3 Admitted
	21	no_treat_v0<br>Show the field ONLY if:<br>[treat_v0] = '0'	Why was treatment NOT given?	notes
	22	cbc_v0	Hemoglobin measured?	yesno<br>1 Yes<br>0 No
	23	hb_v0<br>Show the field ONLY if:<br>[cbc_v0] = '1'	Hemoglobin (g/dL)	text (number, Min: 3, Max: 20)
	24	dbs_v0	Dried blood spots collected?	yesno<br>1 Yes<br>0 No

	25	urine_v0	Urine sample collected?	yesno<br>1 Yes<br>0 No
	26	bednet_mot_v0	Section Header: *IV. Medical History - Mother*<br>Did the mother sleep under a bed net last night?	yesno, Required<br>1 Yes<br>0 No
	27	cosleep_v0<br>Show the field ONLY if:<br>[bednet_mot_v0] = '1'	Did the mother sleep under the same net as the child?	yesno<br>1 Yes<br>0 No
	28	cbc_mot_v0	Section Header: *V. Laboratory Testing - Mother*<br>Hemoglobin measured?	yesno<br>1 Yes<br>0 No
	29	hb_mot_v0<br>Show the field ONLY if:<br>[cbc_mot_v0] = '1'	Hemoglobin (g/dL)	text (number, Min: 3, Max: 20)
	30	dbs_mot_v0	Dried blood spots collected?	yesno<br>1 Yes<br>0 No
	31	urine_mot_v0	Urine sample collected?	yesno<br>1 Yes<br>0 No
	32	dc_v0	Section Header: *VI. Discharge Actions*<br>Prior to discharge, ensure the following are complete:	checkbox<br>0 dc_v0___0 Provide with Study ID card<br>1 dc_v0___1 Give new Lesu according to assignment<br>2 dc_v0___2 Instruct to return to clinic with card if child sick<br>3 dc_v0___3 Remind about next scheduled visit in 2 weeks
	33	initial_visit_complete	Section Header: *Form Status*<br>Complete?	dropdown<br>0 Incomplete<br>1 Unverified<br>2 Complete

Instrument: **Week 2 Visit** (week_2_visit) Collapse

	34	date_visit_1_v2	Section Header: *Week 2 Visit*<br>Date of visit<br>*DD-MM-YYYY*	text (date_dmy), Required
	35	temp_v2	Section Header: *I. Vital Signs - Child*<br>Axillary temperature<br>*degrees Celsius*	text (number, Min: 35, Max: 45), Required
	36	bednet_1_v2	Section Header: *II. Medical History - Child*<br>Did the child sleep under a bed net last night?	yesno, Required<br>1 Yes<br>0 No
	37	fever_1_v2	Has the child had fever in last two weeks?	yesno, Required<br>1 Yes

				0 No
	38	onset_1_v2<br>Show the field ONLY if:<br>[fever_1_v2] = '1'	If yes, when did the fever start<br>*DD-MM-YYYY*	text (date_dmy)
	39	sick_1_v2<br>Show the field ONLY if:<br>[fever_1_v2] = '0'	Even if the child has not had a fever, has he or she been otherwise unwell?	yesno, Required<br>1 Yes<br>0 No
	40	symp_1_v2<br>Show the field ONLY if:<br>[sick_1_v2] = '1'	If yes, what symptoms has the child experienced?	checkbox<br>0 symp_1_v2___0 Cough<br>1 symp_1_v2___1 Diarrhea<br>2 symp_1_v2___2 Ear Ache<br>3 symp_1_v2___3 Not feeding<br>4 symp_1_v2___4 Rash<br>5 symp_1_v2___5 Runny nose<br>6 symp_1_v2___6 Other (specify below)
	41	symp_other_1_v2<br>Show the field ONLY if:<br>[symp_1_v2(6)] = '1'	List other symptoms:	notes
	42	healthcentre_1_v2<br>Show the field ONLY if:<br>[fever_1_v2] = '1' or [sick_1_v2] = '1'	Has the child been seen at a hospital, health centre, clinic, drug shop, or other medical attendant for these symptoms?	yesno<br>1 Yes<br>0 No
	43	hc_where_1_v2<br>Show the field ONLY if:<br>[healthcentre_1_v2] = '1'	If yes, where?	radio<br>0 Hospital<br>1 Health Centre<br>2 Drug Shop or Pharmacy<br>3 VHT<br>4 Traditional Medicine
	44	medicine_1_v2<br>Show the field ONLY if:<br>[healthcentre_1_v2] = '1'	Did the child receive medicine for malaria?	yesno<br>1 Yes<br>0 No
	45	med_date_1_v2<br>Show the field ONLY if:<br>[medicine_1_v2] = '1'	When did the child take the last dose (i.e. pill) of medicine?<br>*DD-MM-YYYY*	text (date_dmy)
	46	mrdt_1_v2	Section Header: *III. Laboratory Testing - Child*<br>Malaria RDT performed?	yesno, Required<br>1 Yes<br>0 No
	47	mrdt_res_1_v2<br>Show the field ONLY if:<br>[mrdt_1_v2] = '1'	Malaria RDT Result<br>*Repeat any invalid tests*	radio<br>0 Negative<br>1 Positive
	48	treat_1_v2<br>Show the field ONLY if:<br>([temp_v2] >= 37.5 or [fever_1 _v2] = '1') and [mrdt_res_1_v2] = '1'	If mother reported fever or child's temperature was >37.5 C, which antimalarial treatment provided?	radio<br>0 None (explain below)<br>1 Coartem<br>2 Quinine<br>3 Admitted

	49	no_treat_1_v2<br>Show the field ONLY if:<br>[treat_1_v2] = '0'	Why was treatment NOT given?	notes
	50	dbs_1_v2	Dried blood spots collected?	yesno<br>1 Yes<br>0 No
	51	lesu_use_2	Section Header: *IV. Lesu Questions*<br>Since your last visit, how often did you use the lesu to carry the child?	radio, Required<br>0 Never<br>1 Some days (1 - 3 per week)<br>2 Most days (4 - 6 per week)<br>3 Every day
	52	washing_2	Since your last visit, how many times did you wash the lesu?	text (integer, Min: 0, Max: 50), Required
	53	se_2	Did the child experience any side effects, to include itching or rash, from the lesu?	yesno, Required<br>1 Yes<br>0 No
	54	se_symp_v2<br>Show the field ONLY if:<br>[se_2] = '1'	If yes, what were the child's side effects?	checkbox<br>0 se_symp_v2___0 Headache<br>1 se_symp_v2___1 Itching<br>2 se_symp_v2___2 Nausea or not feeding<br>3 se_symp_v2___3 Rash<br>4 se_symp_v2___4 Other
	55	se_other_2<br>Show the field ONLY if:<br>[se_symp_v2(4)] = '1'	Describe the child's other symptoms:	notes
	56	se_impact_3<br>Show the field ONLY if:<br>[se_2] = '1'	Did the side effects make you stop using the lesu or use the lesu less frequently?	radio<br>0 No change in use<br>1 Used it less frequently<br>2 Stopped using it
	57	se_moth_2	Did the mother experience any side effects, to include itching or rash, from the lesu?	yesno, Required<br>1 Yes<br>0 No
	58	se_symp_moth_v2<br>Show the field ONLY if:<br>[se_moth_2] = '1'	If yes, what were the side effects?	checkbox<br>0 se_symp_moth_v2___0 Headache<br>1 se_symp_moth_v2___1 Itching<br>2 se_symp_moth_v2___2 Nausea or loss of appetite<br>3 se_symp_moth_v2___3 Rash<br>4 se_symp_moth_v2___4 Other
	59	se_moth_other_2<br>Show the field ONLY if:<br>[se_symp_moth_v2(4)] = '1'	Describe other symptoms:	notes
	60	se_impact_moth_2<br>Show the field ONLY if:<br>[se_moth_2] = '1'	Did these side effects make you stop using the lesu or use the lesu less frequently?	radio<br>0 No change in use<br>1 Used it less frequently

				2 Stopped using it
	61	dc_1_v2	Section Header: *VI. Discharge Actions*<br>Prior to discharge, ensure the following are complete:	checkbox<br>2 dc_1_v2___2 Instruct to return to clinic with card if child sick<br>3 dc_1_v2___3 Remind about next scheduled visit in 2 weeks
	62	week_2_visit_complete	Section Header: *Form Status*<br>Complete?	dropdown<br>0 Incomplete<br>1 Unverified<br>2 Complete
Instrument: **Week 4 Visit** (week_4_visit)				Collapse
	63	date_visit_v4	Section Header: *Week 4 Visit*<br>Date of visit<br>*DD-MM-YYYY*	text (date_dmy), Required
	64	temp_v4	Section Header: *I. Vital Signs - Child*<br>Axillary temperature<br>*degrees Celsius*	text (number, Min: 35, Max: 45), Required
	65	bednet_v4	Section Header: *II. Medical History - Child*<br>Did the child sleep under a bed net last night?	yesno, Required<br>1 Yes<br>0 No
	66	fever_v4	Has the child had fever in last two weeks?	yesno, Required<br>1 Yes<br>0 No
	67	onset_v4<br>Show the field ONLY if:<br>[fever_v4] = '1'	If yes, when did the fever start<br>*DD-MM-YYYY*	text (date_dmy)
	68	sick_v4<br>Show the field ONLY if:<br>[fever_v4] = '0'	Even if the child has not had a fever, has he or she been otherwise unwell?	yesno, Required<br>1 Yes<br>0 No
	69	symp_v4<br>Show the field ONLY if:<br>[sick_v4] = '1'	If yes, what symptoms has the child experienced?	checkbox<br>0 symp_v4___0 Cough<br>1 symp_v4___1 Diarrhea<br>2 symp_v4___2 Ear Ache<br>3 symp_v4___3 Not feeding<br>4 symp_v4___4 Rash<br>5 symp_v4___5 Runny nose<br>6 symp_v4___6 Other (specify below)
	70	symp_other_v4<br>Show the field ONLY if:<br>[symp_v4(6)] = '1'	List other symptoms:	notes
	71	healthcentre_v4<br>Show the field ONLY if:<br>[fever_v4] = '1' or [sick_v4] = '1<br>'	Has the child been seen at a hospital, health centre, clinic, drug shop, or other medical attendant for these symptoms?	yesno<br>1 Yes<br>0 No
	72	hc_where_v4<br>Show the field ONLY if:<br>[healthcentre_v4] = '1'	If yes, where?	radio<br>0 Hospital<br>1 Health Centre

				2 Drug Shop or Pharmacy<br>3 VHT<br>4 Traditional Medicine
	73	medicine_v4<br>Show the field ONLY if:<br>[healthcentre_v4] = '1'	Did the child receive medicine for malaria?	yesno<br>1 Yes<br>0 No
	74	med_date_v4<br>Show the field ONLY if:<br>[medicine_v4] = '1'	When did the child take the last dose (i.e. pill) of medicine?<br>*DD-MM-YYYY*	text (date_dmy)
	75	mrdt_v4	Section Header: *III. Laboratory Testing - Child*<br>Malaria RDT performed?	yesno, Required<br>1 Yes<br>0 No
	76	mrdt_res_v4<br>Show the field ONLY if:<br>[mrdt_v4] = '1'	Malaria RDT Result<br>*Repeat any invalid tests*	radio<br>0 Negative<br>1 Positive
	77	treat_v4<br>Show the field ONLY if:<br>([temp_v4] >= 37.5 or [fever_v4] = '1') and [mrdt_res_v4] = '1'	If mother reported fever or child's temperature was >37.5 C, which antimalarial treatment provided?	radio<br>0 None (explain below)<br>1 Coartem<br>2 Quinine<br>3 Admitted
	78	no_treat_v4<br>Show the field ONLY if:<br>[treat_v4] = '0'	Why was treatment NOT given?	notes
	79	dbs_v4	Dried blood spots collected?	yesno<br>1 Yes<br>0 No
	80	lesu_use_v4	Section Header: *IV. Lesu Questions*<br>Since your last visit, how often did you use the lesu to carry the child?	radio, Required<br>0 Never<br>1 Some days (1 - 3 per week)<br>2 Most days (4 - 6 per week)<br>3 Every day
	81	washing_v4	Since your last visit, how many times did you wash the lesu?	text (integer, Min: 0, Max: 50), Required
	82	se_v4	Did the child experience any side effects, to include itching or rash, from the lesu?	yesno, Required<br>1 Yes<br>0 No
	83	se_symp_v4<br>Show the field ONLY if:<br>[se_v4] = '1'	If yes, what were the child's side effects?	checkbox<br>0 se_symp_v4___0 Headache<br>1 se_symp_v4___1 Itching<br>2 se_symp_v4___2 Nausea or not feeding<br>3 se_symp_v4___3 Rash<br>4 se_symp_v4___4 Other
	84	se_other_v4<br>Show the field ONLY if:	Describe the child's other symptoms:	notes

		[se_symp_v4(4)] = '1'		
	85	se_impact_v4<br>Show the field ONLY if:<br>[se_v4] = '1'	Did the side effects make you stop using the lesu or use the lesu less frequently?	radio<br>0 No change in use<br>1 Used it less frequently<br>2 Stopped using it
	86	se_moth_v4	Did the mother experience any side effects, to include itching or rash, from the lesu?	yesno, Required<br>1 Yes<br>0 No
	87	se_symp_moth_v4<br>Show the field ONLY if:<br>[se_moth_v4] = '1'	If yes, what were the side effects?	checkbox<br>0 se_symp_moth_v4___0 Headache<br>1 se_symp_moth_v4___1 Itching<br>2 se_symp_moth_v4___2 Nausea or loss of appetite<br>3 se_symp_moth_v4___3 Rash<br>4 se_symp_moth_v4___4 Other
	88	se_moth_other_v4<br>Show the field ONLY if:<br>[se_symp_moth_v4(4)] = '1'	Describe other symptoms:	notes
	89	se_impact_moth_v4<br>Show the field ONLY if:<br>[se_moth_v4] = '1'	Did these side effects make you stop using the lesu or use the lesu less frequently?	radio<br>0 No change in use<br>1 Used it less frequently<br>2 Stopped using it
	90	dc_v4	Section Header: *VI. Discharge Actions*<br>Prior to discharge, ensure the following are complete:	checkbox<br>1 dc_v4___1 Retreat lesu according to assignment<br>2 dc_v4___2 Instruct to return to clinic with card if child sick<br>3 dc_v4___3 Remind about next scheduled visit in 2 weeks
	91	week_4_visit_complete	Section Header: *Form Status*<br>Complete?	dropdown<br>0 Incomplete<br>1 Unverified<br>2 Complete

Instrument: **Week 6 Visit** (week_6_visit) Collapse

	92	date_visit_v6	Section Header: *Week 6 Visit*<br>Date of visit<br>*DD-MM-YYYY*	text (date_dmy), Required
	93	temp_v6	Section Header: *I. Vital Signs - Child*<br>Axillary temperature<br>*degrees Celsius*	text (number, Min: 35, Max: 45), Required
	94	bednet_v6	Section Header: *II. Medical History - Child*<br>Did the child sleep under a bed net last night?	yesno, Required<br>1 Yes<br>0 No
	95	fever_v6	Has the child had fever in last two weeks?	yesno, Required<br>1 Yes<br>0 No
	96	onset_v6	If yes, when did the fever start	text (date_dmy)

		Show the field ONLY if: [fever_v6] = '1'	*DD-MM-YYYY*	
	97	sick_v6<br>Show the field ONLY if: [fever_v6] = '0'	Even if the child has not had a fever, has he or she been otherwise unwell?	yesno, Required<br>1 Yes<br>0 No
	98	symp_v6<br>Show the field ONLY if: [sick_v6] = '1'	If yes, what symptoms has the child experienced?	checkbox<br>0 symp_v6___0 Cough<br>1 symp_v6___1 Diarrhea<br>2 symp_v6___2 Ear Ache<br>3 symp_v6___3 Not feeding<br>4 symp_v6___4 Rash<br>5 symp_v6___5 Runny nose<br>6 symp_v6___6 Other (specify below)
	99	symp_other_v6<br>Show the field ONLY if: [symp_v6(6)] = '1'	List other symptoms:	notes
	100	healthcentre_v6<br>Show the field ONLY if: [fever_v6] = '1' or [sick_v6] = '1 '	Has the child been seen at a hospital, health centre, clinic, drug shop, or other medical attendant for these symptoms?	yesno<br>1 Yes<br>0 No
	101	hc_where_v6<br>Show the field ONLY if: [healthcentre_v6] = '1'	If yes, where?	radio<br>0 Hospital<br>1 Health Centre<br>2 Drug Shop or Pharmacy<br>3 VHT<br>4 Traditional Medicine
	102	medicine_v6<br>Show the field ONLY if: [healthcentre_v6] = '1'	Did the child receive medicine for malaria?	yesno<br>1 Yes<br>0 No
	103	med_date_v6<br>Show the field ONLY if: [medicine_v6] = '1'	When did the child take the last dose (i.e. pill) of medicine?<br>*DD-MM-YYYY*	text (date_mdy)
	104	mrdt_v6	Section Header: *III. Laboratory Testing - Child*<br>Malaria RDT performed?	yesno, Required<br>1 Yes<br>0 No
	105	mrdt_res_v6<br>Show the field ONLY if: [mrdt_v6] = '1'	Malaria RDT Result<br>*Repeat any invalid tests*	radio<br>0 Negative<br>1 Positive
	106	treat_v6<br>Show the field ONLY if: ([temp_v6] >= 37.5 or [fever_v6] = '1') and [mrdt_res_v6] = '1'	If mother reported fever or child's temperature was >37.5 C, which antimalarial treatment provided?	radio<br>0 None (explain below)<br>1 Coartem<br>2 Quinine<br>3 Admitted
	107	no_treat_v6<br>Show the field ONLY if:	Why was treatment NOT given?	notes

		[treat_v6] = '0'		
	108	dbs_v6	Dried blood spots collected?	yesno<br>1 Yes<br>0 No
	109	lesu_use_v6	Section Header: *IV. Lesu Questions*<br>Since your last visit, how often did you use the lesu to carry the child?	radio, Required<br>0 Never<br>1 Some days (1 - 3 per week)<br>2 Most days (4 - 6 per week)<br>3 Every day
	110	washing_v6	Since your last visit, how many times did you wash the lesu?	text (integer, Min: 0, Max: 50), Required
	111	se_v6	Did the child experience any side effects, to include itching or rash, from the lesu?	yesno, Required<br>1 Yes<br>0 No
	112	se_symp_v6<br>Show the field ONLY if:<br>[se_v6] = '1'	If yes, what were the child's side effects?	checkbox<br>0 se_symp_v6___0 Headache<br>1 se_symp_v6___1 Itching<br>2 se_symp_v6___2 Nausea or not feeding<br>3 se_symp_v6___3 Rash<br>4 se_symp_v6___4 Other
	113	se_other_v6<br>Show the field ONLY if:<br>[se_symp_v6(4)] = '1'	Describe the child's other symptoms:	notes
	114	se_impact_v6<br>Show the field ONLY if:<br>[se_v6] = '1'	Did the side effects make you stop using the lesu or use the lesu less frequently?	radio<br>0 No change in use<br>1 Used it less frequently<br>2 Stopped using it
	115	se_moth_v6	Did the mother experience any side effects, to include itching or rash, from the lesu?	yesno, Required<br>1 Yes<br>0 No
	116	se_symp_moth_v6<br>Show the field ONLY if:<br>[se_moth_v6] = '1'	If yes, what were the side effects?	checkbox<br>0 se_symp_moth_v6___0 Headache<br>1 se_symp_moth_v6___1 Itching<br>2 se_symp_moth_v6___2 Nausea or loss of appetite<br>3 se_symp_moth_v6___3 Rash<br>4 se_symp_moth_v6___4 Other
	117	se_moth_other_v6<br>Show the field ONLY if:<br>[se_symp_moth_v6(4)] = '1'	Describe other symptoms:	notes
	118	se_impact_moth_v6<br>Show the field ONLY if:<br>[se_moth_v6] = '1'	Did these side effects make you stop using the lesu or use the lesu less frequently?	radio<br>0 No change in use<br>1 Used it less frequently<br>2 Stopped using it
	119	dc_v6	Section Header: *VI. Discharge Actions*	checkbox

			Prior to discharge, ensure the following are complete:	2 dc_v6___2 Instruct to return to clinic with card if child sick<br>3 dc_v6___3 Remind about next scheduled visit in 2 weeks
	120	week_6_visit_complete	Section Header: *Form Status*<br>Complete?	dropdown<br>0 Incomplete<br>1 Unverified<br>2 Complete

Instrument: **Week 8 Visit** (week_8_visit)

Collapse

	121	date_visit_v8	Section Header: *Week 8 Visit*<br>Date of visit<br>*DD-MM-YYYY*	text (date_dmy), Required
	122	temp_v8	Section Header: *I. Vital Signs - Child*<br>Axillary temperature<br>*degrees Celsius*	text (number, Min: 35, Max: 45), Required
	123	bednet_v8	Section Header: *II. Medical History - Child*<br>Did the child sleep under a bed net last night?	yesno, Required<br>1 Yes<br>0 No
	124	fever_v8	Has the child had fever in last two weeks?	yesno, Required<br>1 Yes<br>0 No
	125	onset_v8<br>Show the field ONLY if:<br>[fever_v8] = '1'	If yes, when did the fever start<br>*DD-MM-YYYY*	text (date_dmy)
	126	sick_v8<br>Show the field ONLY if:<br>[fever_v8] = '0'	Even if the child has not had a fever, has he or she been otherwise unwell?	yesno, Required<br>1 Yes<br>0 No
	127	symp_v8<br>Show the field ONLY if:<br>[sick_v8] = '1'	If yes, what symptoms has the child experienced?	checkbox<br>0 symp_v8___0 Cough<br>1 symp_v8___1 Diarrhea<br>2 symp_v8___2 Ear Ache<br>3 symp_v8___3 Not feeding<br>4 symp_v8___4 Rash<br>5 symp_v8___5 Runny nose<br>6 symp_v8___6 Other (specify below)
	128	symp_other_v8<br>Show the field ONLY if:<br>[symp_v8(6)] = '1'	List other symptoms:	notes
	129	healthcentre_v8<br>Show the field ONLY if:<br>[fever_v8] = '1' or [sick_v8] = '1 '	Has the child been seen at a hospital, health centre, clinic, drug shop, or other medical attendant for these symptoms?	yesno<br>1 Yes<br>0 No
	130	hc_where_v8<br>Show the field ONLY if:<br>[healthcentre_v8] = '1'	If yes, where?	radio<br>0 Hospital<br>1 Health Centre<br>2 Drug Shop or Pharmacy

				3 VHT<br>4 Traditional Medicine
	131	medicine_v8<br><br>Show the field ONLY if:<br>[healthcentre_v8] = '1'	Did the child receive medicine for malaria?	yesno<br>1 Yes<br>0 No
	132	med_date_v8<br><br>Show the field ONLY if:<br>[medicine_v8] = '1'	When did the child take the last dose (i.e. pill) of medicine?<br>*DD-MM-YYYY*	text (date_dmy)
	133	mrdt_v8	Section Header: *III. Laboratory Testing - Child*<br>Malaria RDT performed?	yesno, Required<br>1 Yes<br>0 No
	134	mrdt_res_v8<br><br>Show the field ONLY if:<br>[mrdt_v8] = '1'	Malaria RDT Result<br>*Repeat any invalid tests*	radio<br>0 Negative<br>1 Positive
	135	treat_v8<br><br>Show the field ONLY if:<br>([temp_v8] >= 37.5 or [fever_v8] = '1') and [mrdt_res_v8] = '1'	If mother reported fever or child's temperature was >37.5 C, which antimalarial treatment provided?	radio<br>0 None (explain below)<br>1 Coartem<br>2 Quinine<br>3 Admitted
	136	no_treat_v8<br><br>Show the field ONLY if:<br>[treat_v8] = '0'	Why was treatment NOT given?	notes
	137	dbs_v8	Dried blood spots collected?	yesno<br>1 Yes<br>0 No
	138	lesu_use_v8	Section Header: *IV. Lesu Questions*<br>Since your last visit, how often did you use the lesu to carry the child?	radio, Required<br>0 Never<br>1 Some days (1 - 3 per week)<br>2 Most days (4 - 6 per week)<br>3 Every day
	139	washing_v8	Since your last visit, how many times did you wash the lesu?	text (integer, Min: 0, Max: 50), Required
	140	se_v8	Did the child experience any side effects, to include itching or rash, from the lesu?	yesno, Required<br>1 Yes<br>0 No
	141	se_symp_v8<br><br>Show the field ONLY if:<br>[se_v8] = '1'	If yes, what were the child's side effects?	checkbox<br>0 se_symp_v8___0 Headache<br>1 se_symp_v8___1 Itching<br>2 se_symp_v8___2 Nausea or not feeding<br>3 se_symp_v8___3 Rash<br>4 se_symp_v8___4 Other
	142	se_other_v8<br><br>Show the field ONLY if:<br>[se_symp_v8(4)] = '1'	Describe the child's other symptoms:	notes
	143	se_impact_v8	Did the side effects make you stop using the lesu or use	radio

		Show the field ONLY if: [se_v8] = '1'	the lesu less frequently?	0 No change in use<br>1 Used it less frequently<br>2 Stopped using it
	144	se_moth_v8	Did the mother experience any side effects, to include itching or rash, from the lesu?	yesno, Required<br>1 Yes<br>0 No
	145	se_symp_moth_v8<br>Show the field ONLY if: [se_moth_v8] = '1'	If yes, what were the side effects?	checkbox<br>0 se_symp_moth_v8___0 Headache<br>1 se_symp_moth_v8___1 Itching<br>2 se_symp_moth_v8___2 Nausea or loss of appetite<br>3 se_symp_moth_v8___3 Rash<br>4 se_symp_moth_v8___4 Other
	146	se_moth_other_v8<br>Show the field ONLY if: [se_symp_moth_v8(4)] = '1'	Describe other symptoms:	notes
	147	se_impact_moth_v8<br>Show the field ONLY if: [se_moth_v8] = '1'	Did these side effects make you stop using the lesu or use the lesu less frequently?	radio<br>0 No change in use<br>1 Used it less frequently<br>2 Stopped using it
	148	dc_v8	Section Header: *VI. Discharge Actions*<br>Prior to discharge, ensure the following are complete:	checkbox<br>1 dc_v8___1 Retreat lesu according to assignment<br>2 dc_v8___2 Instruct to return to clinic with card if child sick<br>3 dc_v8___3 Remind about next scheduled visit in 2 weeks
	149	week_8_visit_complete	Section Header: *Form Status*<br>Complete?	dropdown<br>0 Incomplete<br>1 Unverified<br>2 Complete

Instrument: **Week 10 Visit** (week_10_visit) Collapse

	150	date_visit_v10	Section Header: *Week 10 Visit*<br>Date of visit<br>*DD-MM-YYYY*	text (date_dmy), Required
	151	temp_v10	Section Header: *I. Vital Signs - Child*<br>Axillary temperature<br>*degrees Celsius*	text (number, Min: 35, Max: 45), Required
	152	bednet_v10	Section Header: *II. Medical History - Child*<br>Did the child sleep under a bed net last night?	yesno, Required<br>1 Yes<br>0 No
	153	fever_v10	Has the child had fever in last two weeks?	yesno, Required<br>1 Yes<br>0 No
	154	onset_v10<br>Show the field ONLY if: [fever_v10] = '1'	If yes, when did the fever start<br>*DD-MM-YYYY*	text (date_dmy)

	155	sick_v10<br>Show the field ONLY if:<br>[fever_v10] = '0'	Even if the child has not had a fever, has he or she been otherwise unwell?	yesno, Required<br>1 Yes<br>0 No
	156	symp_v10<br>Show the field ONLY if:<br>[sick_v10] = '1'	If yes, what symptoms has the child experienced?	checkbox<br>0 symp_v10___0 Cough<br>1 symp_v10___1 Diarrhea<br>2 symp_v10___2 Ear Ache<br>3 symp_v10___3 Not feeding<br>4 symp_v10___4 Rash<br>5 symp_v10___5 Runny nose<br>6 symp_v10___6 Other (specify below)
	157	symp_other_v10<br>Show the field ONLY if:<br>[symp_v10(6)] = '1'	List other symptoms:	notes
	158	healthcentre_v10<br>Show the field ONLY if:<br>[fever_v10] = '1' or [sick_v10] = '1'	Has the child been seen at a hospital, health centre, clinic, drug shop, or other medical attendant for these symptoms?	yesno<br>1 Yes<br>0 No
	159	hc_where_v10<br>Show the field ONLY if:<br>[healthcentre_v10] = '1'	If yes, where?	radio<br>0 Hospital<br>1 Health Centre<br>2 Drug Shop or Pharmacy<br>3 VHT<br>4 Traditional Medicine
	160	medicine_v10<br>Show the field ONLY if:<br>[healthcentre_v10] = '1'	Did the child receive medicine for malaria?	yesno<br>1 Yes<br>0 No
	161	med_date_v10<br>Show the field ONLY if:<br>[medicine_v10] = '1'	When did the child take the last dose (i.e. pill) of medicine?<br>*DD-MM-YYYY*	text (date_dmy)
	162	mrdt_v10	Section Header: *III. Laboratory Testing - Child*<br>Malaria RDT performed?	yesno, Required<br>1 Yes<br>0 No
	163	mrdt_res_v10<br>Show the field ONLY if:<br>[mrdt_v10] = '1'	Malaria RDT Result<br>*Repeat any invalid tests*	radio<br>0 Negative<br>1 Positive
	164	treat_v10<br>Show the field ONLY if:<br>([temp_v10] >= 37.5 or [fever_v10] = '1') and [mrdt_res_v10] = '1'	If mother reported fever or child's temperature was >37.5 C, which antimalarial treatment provided?	radio<br>0 None (explain below)<br>1 Coartem<br>2 Quinine<br>3 Admitted
	165	no_treat_v10<br>Show the field ONLY if:<br>[treat_v10] = '0'	Why was treatment NOT given?	notes
	166	dbs_v10	Dried blood spots collected?	yesno

				1 Yes<br>0 No
	167	lesu_use_v10	Section Header: *IV. Lesu Questions*<br>Since your last visit, how often did you use the lesu to carry the child?	radio, Required<br>0 Never<br>1 Some days (1 - 3 per week)<br>2 Most days (4 - 6 per week)<br>3 Every day
	168	washing_v10	Since your last visit, how many times did you wash the lesu?	text (integer, Min: 0, Max: 50), Required
	169	se_v10	Did the child experience any side effects, to include itching or rash, from the lesu?	yesno, Required<br>1 Yes<br>0 No
	170	se_symp_v10<br>Show the field ONLY if:<br>[se_v10] = '1'	If yes, what were the child's side effects?	checkbox<br>0 se_symp_v10___0 Headache<br>1 se_symp_v10___1 Itching<br>2 se_symp_v10___2 Nausea or not feeding<br>3 se_symp_v10___3 Rash<br>4 se_symp_v10___4 Other
	171	se_other_v10<br>Show the field ONLY if:<br>[se_symp_v10(4)] = '1'	Describe the child's other symptoms:	notes
	172	se_impact_v10<br>Show the field ONLY if:<br>[se_v10] = '1'	Did the side effects make you stop using the lesu or use the lesu less frequently?	radio<br>0 No change in use<br>1 Used it less frequently<br>2 Stopped using it
	173	se_moth_v10	Did the mother experience any side effects, to include itching or rash, from the lesu?	yesno, Required<br>1 Yes<br>0 No
	174	se_symp_moth_v10<br>Show the field ONLY if:<br>[se_moth_v10] = '1'	If yes, what were the side effects?	checkbox<br>0 se_symp_moth_v10___0 Headache<br>1 se_symp_moth_v10___1 Itching<br>2 se_symp_moth_v10___2 Nausea or loss of appetite<br>3 se_symp_moth_v10___3 Rash<br>4 se_symp_moth_v10___4 Other
	175	se_moth_other_v10<br>Show the field ONLY if:<br>[se_symp_moth_v10(4)] = '1'	Describe other symptoms:	notes
	176	se_impact_moth_v10<br>Show the field ONLY if:<br>[se_moth_v10] = '1'	Did these side effects make you stop using the lesu or use the lesu less frequently?	radio<br>0 No change in use<br>1 Used it less frequently<br>2 Stopped using it
	177	dc_v10	Section Header: *VI. Discharge Actions*<br>Prior to discharge, ensure the following are complete:	checkbox<br>2 dc_v10___2 Instruct to return to clinic with card if child sick

				3 | dc_v10___3 | Remind about next scheduled visit in 2 weeks
	178	week_10_visit_complete	Section Header: *Form Status*<br>Complete?	dropdown<br>0 | Incomplete<br>1 | Unverified<br>2 | Complete

Instrument: **Week 12 Visit** (week_12_visit)

	179	date_visit_v12	Section Header: *Week 12 (Final) Visit*<br>Date of visit<br>*DD-MM-YYYY*	text (date_dmy), Required
	180	height_v12	Section Header: *I. Vital Signs - Child*<br>Height<br>*cm*	text (number, Min: 50, Max: 100), Required
	181	weight_v12	Weight<br>*kg*	text (number, Min: 5, Max: 20), Required
	182	muac_v12	Mid-Upper Arm Circumference<br>*cm*	text (number, Min: 5, Max: 25), Required
	183	temp_v12	Axillary temperature<br>*degrees Celsius*	text (number, Min: 35, Max: 45), Required
	184	bednet_v12	Section Header: *II. Medical History - Child*<br>Did the child sleep under a bed net last night?	yesno, Required<br>1 | Yes<br>0 | No
	185	fever_v12	Has the child had fever in last two weeks?	yesno, Required<br>1 | Yes<br>0 | No
	186	onset_v12<br>Show the field ONLY if:<br>[fever_v12] = '1'	If yes, when did the fever start<br>*DD-MM-YYYY*	text (date_dmy)
	187	sick_v12<br>Show the field ONLY if:<br>[fever_v12] = '0'	Even if the child has not had a fever, has he or she been otherwise unwell?	yesno, Required<br>1 | Yes<br>0 | No
	188	symp_v12<br>Show the field ONLY if:<br>[sick_v12] = '1'	If yes, what symptoms has the child experienced?	checkbox<br>0 | symp_v12___0 | Cough<br>1 | symp_v12___1 | Diarrhea<br>2 | symp_v12___2 | Ear Ache<br>3 | symp_v12___3 | Not feeding<br>4 | symp_v12___4 | Rash<br>5 | symp_v12___5 | Runny nose<br>6 | symp_v12___6 | Other (specify below)
	189	symp_other_v12<br>Show the field ONLY if:<br>[symp_v12(6)] = '1'	List other symptoms:	notes
	190	healthcentre_v12<br>Show the field ONLY if:<br>[fever_v12] = '1' or [sick_v12] = '1'	Has the child been seen at a hospital, health centre, clinic, drug shop, or other medical attendant for these symptoms?	yesno<br>1 | Yes<br>0 | No
	191	hc_where_v12	If yes, where?	radio<br>0 | Hospital

		Show the field ONLY if: [healthcentre_v12] = '1'		1 Health Centre<br>2 Drug Shop or Pharmacy<br>3 VHT<br>4 Traditional Medicine
	192	medicine_v12<br>Show the field ONLY if: [healthcentre_v12] = '1'	Did the child receive medicine for malaria?	yesno<br>1 Yes<br>0 No
	193	med_date_v12<br>Show the field ONLY if: [medicine_v12] = '1'	When did the child take the last dose (i.e. pill) of medicine?<br>*DD-MM-YYYY*	text (date_dmy)
	194	mrdt_v12	Section Header: *III. Laboratory Testing - Child*<br>Malaria RDT performed?	yesno, Required<br>1 Yes<br>0 No
	195	mrdt_res_v12<br>Show the field ONLY if: [mrdt_v12] = '1'	Malaria RDT Result<br>*Repeat any invalid tests*	radio<br>0 Negative<br>1 Positive
	196	treat_v12<br>Show the field ONLY if: ([temp_v12] >= 37.5 or [fever_v12] = '1') and [mrdt_res_v12] = '1'	If mother reported fever or child's temperature was >37.5 C, which antimalarial treatment provided?	radio<br>0 None (explain below)<br>1 Coartem<br>2 Quinine<br>3 Admitted
	197	no_treat_v12<br>Show the field ONLY if: [treat_v12] = '0'	Why was treatment NOT given?	notes
	198	cbc_v12	Hemoglobin measured?	yesno<br>1 Yes<br>0 No
	199	hb_v12<br>Show the field ONLY if: [cbc_v12] = '1'	Hemoglobin (g/dL)	text (number, Min: 3, Max: 20)
	200	dbs_v12	Dried blood spots collected?	yesno<br>1 Yes<br>0 No
	201	urine_v12	Urine sample collected?	yesno<br>1 Yes<br>0 No
	202	bednet_mot_v12	Section Header: *IV. Medical History - Mother*<br>Did the mother sleep under a bed net last night?	yesno, Required<br>1 Yes<br>0 No
	203	cosleep_v12<br>Show the field ONLY if: [bednet_mot_v12] = '1'	Did the mother sleep under the same net as the child?	yesno<br>1 Yes<br>0 No
	204	cbc_mot_v12	Section Header: *V. Laboratory Testing - Mother*<br>Hemoglobin measured?	yesno<br>1 Yes

				0 No
	205	hb_mot_v12<br>Show the field ONLY if:<br>[cbc_mot_v12] = '1'	Hemoglobin (g/dL)	text (number, Min: 3, Max: 20)
	206	dbs_mot_v12	Dried blood spots collected?	yesno<br>1 Yes<br>0 No
	207	urine_mot_v12	Urine sample collected?	yesno<br>1 Yes<br>0 No
	208	dc_v12	Section Header: *VI. Discharge Actions*<br>Prior to discharge, ensure the following are complete:	checkbox<br>0 dc_v12___0 Complete Exit Interview<br>1 dc_v12___1 Give completion bonus (if eligible)<br>2 dc_v12___2 Thank for participation in the study
	209	week_12_visit_complete	Section Header: *Form Status*<br>Complete?	dropdown<br>0 Incomplete<br>1 Unverified<br>2 Complete

Instrument: **Exit Interview** (exit_interview) Collapse

	210	exit_date	Date	text (date_dmy), Required
	211	exit_use	How did you use the lesu?<br>*Check all that apply*	checkbox, Required<br>0 exit_use___0 Carry child on back<br>1 exit_use___1 Blanket for sleep<br>2 exit_use___2 Cloth to set child down on ground<br>3 exit_use___3 Other<br>Custom alignment: RH
	212	exit_use_other	If other, please describe	notes<br>Custom alignment: RH
	213	exit_lessmore	Dod you use this lesu more or less than lesus with your other children?	dropdown, Required<br>0 Less<br>1 More<br>2 Not applicable - this was first child<br>Custom alignment: RH
	214	exit_lessmore_des<br>Show the field ONLY if:<br>[exit_lessmore] = '0' or [exit_lessmore] = '1'	Why did you use it less or more?	notes<br>Custom alignment: RH
	215	exit_se	Did you observe your child having any side effects from the lesu over the study period?	yesno, Required<br>1 Yes<br>0 No
	216	exit_se_des<br>Show the field ONLY if:<br>[exit_se] = '1'	If yes, please describe:	notes

	217	exit_mat_se	Did you have any side effects from wearing the lesu over the study period?	yesno, Required<br>1 Yes<br>0 No
	218	exit_mat_se_des<br>Show the field ONLY if:<br>[exit_mat_se] = '1'	If yes, please describe:	notes
	219	exit_ben	Did you observe any benefit against mosquitos and other bugs from the lesu?	yesno, Required<br>1 Yes<br>0 No
	220	exit_ben_des<br>Show the field ONLY if:<br>[exit_ben] = '1'	If yes, please describe:	notes
	221	exit_rec	Would you recommend lesus treated with insecticide to a family member or friend?	yesno<br>1 Yes<br>0 No
	222	exit_rec_des	Please explain why or why not?	notes
	223	exit_pay	Would you be willing to pay more for a lesu that was treated with insecticide?	yesno, Required<br>1 Yes<br>0 No
	224	exit_pay_amt<br>Show the field ONLY if:<br>[exit_pay] = '1'	How much more would you be willing to pay for a treated lesu than a standard lesu?	dropdown<br>0 1,000 shillings<br>1 2,500 shillings<br>2 5,000 shillings<br>3 10,000 shillings<br>4 >10,000 shillings<br>Custom alignment: RH
	225	exit_final	Do you have any final comments about the lesu?	notes, Required<br>Custom alignment: RH
	226	exit_interview_complete	Section Header: *Form Status*<br>Complete?	dropdown<br>0 Incomplete<br>1 Unverified<br>2 Complete

Instrument: **Home Visit** (home_visit_d08c3f) Collapse

	227	time	Section Header: *Section 1: Respondent's Background*<br>101. RECORD THE TIME	text (time)
	228	birth_date	102. In what month and year were you born?	text (date_dmy), Identifier
	229	birth_date_unknown	If unknown, select:	checkbox<br>1 birth_date_unknown___1 Don't know month<br>2 birth_date_unknown___2 Dont know year
	230	age_years	103. How old were you at your last birthday?<br>*age in completed years*	text (number, Min: 18, Max: 65), Identifier
	231	school_yn	104. Have you ever attended school?	yesno<br>1 Yes<br>0 No
	232	school_highestlevel	105. What is the highest level of school you attended?	radio

		Show the field ONLY if: [school_yn] = '1'		1 Primary<br>2 'O' Level<br>3 'A' Level<br>4 University/Tertiary
	233	school_highestyear<br>Show the field ONLY if: [school_yn] = '1'	106. What is the highest (class/year) you completed at that level?<br><br>IF COMPLETED LESS THAN ONE YEAR AT THAT LEVEL, RECORD '00'.<br>*if less than one year, record "00"*	text (number, Min: 0)
	234	school_level<br>Show the field ONLY if: [school_yn]='1'	107. CHECK 105:	radio<br>1 PRIMARY<br>2 SECONDARY OR HIGHER
	235	read_card	108. Now I would like you to read this sentence to me.<br><br>SHOW CARD TO RESPONDENT.<br><br>IF RESPONDENT CANNOT READ WHOLE SENTENCE, PROBE:<br><br>Can you read any part of the sentence to me?	radio<br>1 Cannot read at all<br>2 Able to read only parts of sentence<br>3 Able to read whole sentence<br>4 No card with required language<br>5 Blind/Visually impaired
	236	language<br>Show the field ONLY if: [read_card] = '4'	Please specify the language	notes
	237	religion	109. What is your religion?	radio<br>1 Catholic<br>2 Anglican/Protestant<br>3 SDA<br>4 Pentecostal<br>5 Other Christian<br>6 Moslem<br>7 Bahai<br>8 Traditional<br>9 Hindu<br>10 None<br>99 Other
	238	religion_other<br>Show the field ONLY if: [religion] = '99'	Specify Religion	notes
	239	ethnicity	110. What is your ethnic group?	radio<br>1 Baganda<br>2 Banyankore<br>3 Iteso<br>4 Lugbara/Madi<br>5 Basoga<br>6 Langi<br>7 Bakiga<br>8 Karimojong

				9 Acholi<br>10 Bagisu/Sabiny<br>11 Alur/Jopadhola<br>12 Banyoro<br>13 Batoro<br>99 Other
	240	ethnicity_other<br>Show the field ONLY if:<br>[ethnicity] = '99'	Specify ethnic group	notes
	241	messages_yn	111. In the past six months, have you seen or heard any messages about malaria?	yesno<br>1 Yes<br>0 No
	242	messages_where<br>Show the field ONLY if:<br>[messages_yn] = '1'	112. Have you seen or heard these messages:	checkbox<br>1 messages_where___1 On the radio<br>2 messages_where___2 On the television<br>3 messages_where___3 On a poster or billboard<br>4 messages_where___4 From a community health worker<br>5 messages_where___5 At a community event<br>6 messages_where___6 Anywhere else
	243	birth_any_yn	Section Header: *Section 2: Reproduction*<br>201. Now I would like to ask about all the births you have had during your life. Have you ever given birth?	yesno<br>1 Yes<br>0 No
	244	kids_home_yn<br>Show the field ONLY if:<br>[birth_any_yn] = '1'	202. Do you have any sons or daughters to whom you have given birth who are now living with you?	yesno<br>1 Yes<br>0 No
	245	sons_home<br>Show the field ONLY if:<br>[kids_home_yn] = '1'	203 (a). How many sons live with you?<br><br>IF NONE, RECORD 00	text (integer, Min: 0)
	246	daughters_home<br>Show the field ONLY if:<br>[kids_home_yn] = '1'	203 (b) And how many daughters live with you?<br><br>IF NONE, RECORD '00'.	text (integer, Min: 0)
	247	kids_elsewhere_yn	204. Do you have any sons or daughters to whom you have given birth who are alive but do not live with you?	yesno<br>1 Yes<br>0 No
	248	sons_elsewhere<br>Show the field ONLY if:<br>[kids_elsewhere_yn] = '1'	205 (a) How many sons are alive but do not live with you?<br><br>IF NONE, RECORD '00'.	text (number, Min: 0)
	249	daughters_elsewhere_acc7c3<br>Show the field ONLY if:<br>[kids_elsewhere_yn] = '1'	205 (b) And how many daughters are alive but do not live with you?<br><br>IF NONE, RECORD '00'.	text (number, Min: 0)
	250	birth_laterdied_yn	206. Have you ever given birth to a boy or girl who was born alive but later died?	yesno<br>1 Yes<br>0 No
	251	sons_laterdied	207(a). How many boys have died?	text (integer, Min: 0)

		Show the field ONLY if: [birth_laterdied_yn] = '1'	IF NONE, RECORD '00'.	
	252	daughters_laterdied<br>Show the field ONLY if: [birth_laterdied_yn] = '1'	207(b). And how many girls have died?<br><br>IF NONE, RECORD '00'.	text (integer, Min: 0)
	253	birth_notsurvived_yn<br>Show the field ONLY if: [birth_laterdied_yn] = '0'	Any baby who cried or showed signs of life but did not survive?	yesno<br>1 Yes<br>0 No
	254	birth_total	208. SUM ANSWERS TO 203, 205 AND 207. AND ENTER TOTAL BIRTHS.<br><br>IF NONE, RECORD '00'.	text (integer, Min: 0)
	255	check_208	209. CHECK 208!	descriptive
	256	mrb_number	210. Now I'd like to ask you about your more recent births. How many births have you had in the last 6 years?<br><br>IF NONE, RECORD '00'.	text (integer, Min: 0, Max: 10)
	257	check_215	Section Header: *Section 3: Pregnancy and Intermittent Preventative Treatment*<br>301. CHECK 215. ENTER IN THE TABLE THE NAME AND SURVIVAL STATUS OF THE MOST RECENT BIRTH.<br><br>Now I would like to ask you some questions about your last pregnancy that resulted in a live birth.	descriptive
	258	mrb_name	301(a). MOST RECENT BIRTH--NAME	notes, Identifier
	259	mrb_livingordead	301(b). MOST RECENT BIRTH--Living or Dead?	radio<br>1 Living<br>2 Dead
	260	antenatal_yn	302. When you were pregnant with (NAME), did you see anyone for antenatal care for this pregnancy?	yesno<br>1 Yes<br>0 No
	261	antenatal_provider	303. Whom did you see?<br><br>Anyone else?	radio<br>1 Doctor<br>2 Nurse/Midwife<br>3 Medical Assistant/Clinical Officer<br>4 Nursing Aide<br>5 Traditional Birth Attendant<br>6 Other
	262	antenatal_provider_other<br>Show the field ONLY if: [antenatal_provider] = '6'	303. Specify:	notes
	263	antenatal_whynone<br>Show the field ONLY if: [antenatal_yn] = '0'	303(a). What was the main reason why you did not see anyone for antenatal care?	radio<br>1 Clinic too far<br>2 Had no money<br>3 Had no time<br>4 Not aware had to attend<br>5 Did not want to attend<br>6 Other<br>8 Don't know

	264	antenatal_whynone_other<br>Show the field ONLY if:<br>[antenatal_whynone] = '6'	303(a). Specify:	notes
	265	pregnancy_malariadrug_yn	304. During this pregnancy, did you take any drugs to keep you from getting malaria?	radio<br>1 Yes<br>0 No<br>8 Don't know
	266	pregnancy_malariadrug_whynone<br>Show the field ONLY if:<br>[pregnancy_malariadrug_yn] = '0'	304(a). What was the main reason why you did not take any drugs to keep you from getting malaria during this pregnancy?	radio<br>1 Clinic too far<br>2 Had no money<br>3 Side effects<br>4 Not aware had to take any<br>5 Did not want to take<br>6 Other<br>8 Don't know
	267	pregnancy_malariadrug_whynone_other<br>Show the field ONLY if:<br>[pregnancy_malariadrug_whynone] = '6'	304(a). Specify:	notes
	268	pregnancy_malariadrug	305. What drugs did you take?<br><br>RECORD ALL MENTIONED<br><br>IF TYPE OF DRUG IS NOT DETERMINED, SHOW TYPICAL ANTIMALARIAL DRUGS TO RESPONDENT.	checkbox<br>1 pregnancy_malariadrug___1 SP/Fansidar<br>2 pregnancy_malariadrug___2 Chloroquine<br>3 pregnancy_malariadrug___3 Other<br>4 pregnancy_malariadrug___4 Don't know
	269	pregnancy_spfansidar_freq<br>Show the field ONLY if:<br>[pregnancy_malariadrug(1)] = '1'	307. How many times did you take SP/Fansidar during this pregnancy?	text (integer, Min: 0)
	270	pregnancy_spfansidar_whyonce<br>Show the field ONLY if:<br>[pregnancy_spfansidar_freq] < 2	307(b). Why did you take (SP/Fansidar) only one time during this pregnancy?	radio<br>1 Clinic too far<br>2 Had no money<br>3 Side effects<br>4 Not aware had to take more<br>5 Did not want to take<br>6 Other<br>7 Don't know
	271	pregnancy_spfansidar_whyonce_other<br>Show the field ONLY if:<br>[pregnancy_spfansidar_whyonce] = '6'	307(b). Specify:	notes
	272	pregnancy_spfansidar_where<br>Show the field ONLY if:<br>[pregnancy_malariadrug(1)] = '1'	309. Did you get the (SP/Fansidar) during any antenatal care visit, during another visit to a health facility or from another source?	radio<br>1 Antenatal visit<br>2 Another facility visit<br>3 Other source

	273	check_215_216	310: CHECK 215 AND 216:	radio<br>1 ONE OR MORE LIVING CHILDREN BORN IN 2008 OR LATER<br>2 NO LIVING CHILDREN BORN IN 2008 OR LATER
	274	check_215_401<br>Show the field ONLY if:<br>[check_215_216] = '1'	Section Header: *Section 4: Fever in Children*<br>401. CHECK 215: ENTER IN THE TABLE THE BIRTH HISTORY NUMBER, NAME, AND SURVIVAL STATUS OF EACH BIRTH IN 2014 OR LATER. ASK THE QUESTIONS ABOUT ALL OF THESE BIRTHS. BEGIN WITH THE MOST RECENT BIRTH. IF MORE THAN THREE BIRTHS-USE AN ADDITIONAL QUESTIONNAIRE.	descriptive
	275	mrb_birth_number<br>Show the field ONLY if:<br>[check_215_216] = '1'	402. MOST RECENT BIRTH<br><br>Birth history number from 212 in birth history:	text
	276	mrb_name2<br>Show the field ONLY if:<br>[mrb_birth_number] >= 00	403. FROM 212 AND 216<br><br>Name:	text, Identifier
	277	mrb_livingordead2<br>Show the field ONLY if:<br>[mrb_birth_number] >= 00	403(a). Is (name) living or deceased?	radio<br>1 Living<br>2 Dead
	278	mrb_fever_yn<br>Show the field ONLY if:<br>[mrb_livingordead2] = '1'	404. Has (Name) been ill with a fever at any time in the last 2 weeks?	radio<br>1 Yes<br>0 No<br>99 Don't Know
	279	mrb_blooddraw_yn<br>Show the field ONLY if:<br>[mrb_fever_yn] = '1'	405. At any time during the illness, did (NAME) have blood taken from his/her finger or heel for testing?	radio<br>1 Yes<br>2 No<br>3 Don't know
	280	mrb_adviceortrt_yn<br>Show the field ONLY if:<br>[mrb_fever_yn]='1'	406. Did you seek advice or treatment for the illness from any source?	radio<br>1 Yes<br>0 No
	281	mrb_adviceortrt_whynone<br>Show the field ONLY if:<br>[mrb_adviceortrt_yn] = '0'	406(a). Why have you not sought advice or treatment from any source?	radio<br>1 Child just fell ill<br>2 Child not very ill<br>3 Clinic too far<br>4 Have no money<br>5 Waiting for child's father<br>6 Don't know what to do<br>7 Already had medicine at home<br>8 Other
	282	mrb_adviceortrt_whynone_other<br>Show the field ONLY if:<br>[mrb_adviceortrt_yn] = '8'	406(a). Specify.	notes
	283	mrb_adviceortrt_where<br>Show the field ONLY if:<br>[mrb_adviceortrt_yn] = '1'	407. Where did you seek advice or treatment? Anywhere else?	checkbox<br>1 mrb_adviceortrt_where___1 Government hospital

				2 mrb_adviceortrt_where___2 Government health center<br>3 mrb_adviceortrt_where___3 Government health post<br>4 mrb_adviceortrt_where___4 Mobile clinic<br>5 mrb_adviceortrt_where___5 Community health worker (VHT)<br>6 mrb_adviceortrt_where___6 Other public sector<br>7 mrb_adviceortrt_where___7 Public sector (PNFP) hospital<br>8 mrb_adviceortrt_where___8 Public sector (PNFP) health center<br>9 mrb_adviceortrt_where___9 Private hospital/clinic<br>10 mrb_adviceortrt_where___10 Pharmacy<br>11 mrb_adviceortrt_where___11 Private mobile clinic<br>12 mrb_adviceortrt_where___12 Fieldworker<br>13 mrb_adviceortrt_where___13 Other private medical sector<br>14 mrb_adviceortrt_where___14 Shop<br>15 mrb_adviceortrt_where___15 Traditional practitioner<br>16 mrb_adviceortrt_where___16 Market<br>17 mrb_adviceortrt_where___17 Other
	284	mrb_adviceortrt_where_other<br>Show the field ONLY if:<br>[mrb_adviceortrt_yn]='1'	407(a). Specify, and/or if unable to determine if public or private sector, write the name of the place(s).	notes
	285	mrb_check407	408. CHECK 407:	radio<br>1 TWO OR MORE CODES CIRCLED<br>2 ONLY ONE CODE CIRCLED
	286	mrb_adviceortrt_first_where<br>Show the field ONLY if:<br>[mrb_check407] = '1'	409. Where did you first seek advice or treatment? USE LETTER CODE FROM 407.<br><br>FIRST PLACE:	text
	287	mrb_drugstaken_yn	410. At any time during the illness, did (NAME) take any drugs for the illness?	radio<br>1 Yes<br>2 No<br>3 Don't know
	288	smrb_yn	Is there a second most recent birth?	yesno<br>1 Yes<br>0 No
	289	smrb_birth_number<br>Show the field ONLY if:<br>[smrb_yn] = '1'	402. SECOND MOST RECENT BIRTH<br><br>Birth history number from 212 in birth history:	text
	290	smrb_name	403. FROM 212 AND 216	text, Identifier

		Show the field ONLY if: [smrb_birth_number] >= 00	Name:	
	291	smrb_livingordead<br>Show the field ONLY if: [smrb_birth_number] >= 00	403(a). Is (name) living or deceased?	radio<br>1 Living<br>2 Dead
	292	smrb_fever_yn<br>Show the field ONLY if: [smrb_livingordead] = '1'	404. Has (Name) been ill with a fever at any time in the last 2 weeks?	radio<br>1 Yes<br>0 No<br>99 Don't Know
	293	smrb_blooddraw_yn<br>Show the field ONLY if: [smrb_fever_yn] = '1'	405. At any time during the illness, did (NAME) have blood taken from his/her finger or heel for testing?	radio<br>1 Yes<br>2 No<br>3 Don't know
	294	smrb_adviceortrt_yn<br>Show the field ONLY if: [smrb_fever_yn] = '1'	406. Did you seek advice or treatment for the illness from any source?	radio<br>1 Yes<br>0 No
	295	smrb_adviceortrt_whynone<br>Show the field ONLY if: [smrb_adviceortrt_yn] = '0'	406(a). Why have you not sought advice or treatment from any source?	radio<br>1 Child just fell ill<br>2 Child not very ill<br>3 Clinic too far<br>4 Have no money<br>5 Waiting for child's father<br>6 Don't know what to do<br>7 Already had medicine at home<br>8 Other
	296	smrb_adviceortrt_whynone_other<br>Show the field ONLY if: [smrb_adviceortrt_whynone] = '8'	406(a). Specify.	notes
	297	smrb_adviceortrt_where<br>Show the field ONLY if: [smrb_adviceortrt_yn] = '1'	407. Where did you seek advice or treatment? Anywhere else?	checkbox<br>1 smrb_adviceortrt_where___1 Government hospital<br>2 smrb_adviceortrt_where___2 Government health center<br>3 smrb_adviceortrt_where___3 Government health post<br>4 smrb_adviceortrt_where___4 Mobile clinic<br>5 smrb_adviceortrt_where___5 Community health worker (VHT)<br>6 smrb_adviceortrt_where___6 Other public sector<br>7 smrb_adviceortrt_where___7 Public sector (PNFP) hospital<br>8 smrb_adviceortrt_where___8 Public sector (PNFP) health center

				9 smrb_adviceortrt_where___9 Private hospital/clinic<br>10 smrb_adviceortrt_where___10 Pharmacy<br>11 smrb_adviceortrt_where___11 Private mobile clinic<br>12 smrb_adviceortrt_where___12 Fieldworker<br>13 smrb_adviceortrt_where___13 Other private medical sector<br>14 smrb_adviceortrt_where___14 Shop<br>15 smrb_adviceortrt_where___15 Traditional practitioner<br>16 smrb_adviceortrt_where___16 Market<br>17 smrb_adviceortrt_where___17 Other
	298	smrb_adviceortrt_where_other<br>Show the field ONLY if:<br>[smrb_adviceortrt_yn]='1'	407(a). Specify, and/or if unable to determine if public or private sector, write the name of the place(s).	notes
	299	smrb_check407<br>Show the field ONLY if:<br>[smrb_adviceortrt_yn]='1'	408. CHECK 407:	radio<br>1 TWO OR MORE CODES CIRCLED<br>2 ONLY ONE CODE CIRCLED
	300	smrb_adviceortrt_first_where<br>Show the field ONLY if:<br>[smrb_check407] = '1'	409. Where did you first seek advice or treatment? USE LETTER CODE FROM 407.<br><br>FIRST PLACE:	text
	301	smrb_drugstaken_yn<br>Show the field ONLY if:<br>[smrb_adviceortrt_yn]='1'	410. At any time during the illness, did (NAME) take any drugs for the illness?	radio<br>1 Yes<br>0 No<br>99 Don't know
	302	tmrb_yn	Is there a third most recent birth?	yesno<br>1 Yes<br>0 No
	303	tmrb_birth_number<br>Show the field ONLY if:<br>[tmrb_yn] = '1'	402. THIRD MOST RECENT BIRTH<br><br>Birth history number from 212 in birth history:	text
	304	tmrb_name<br>Show the field ONLY if:<br>[tmrb_yn] >= 00	403. FROM 212 AND 216<br><br>Name:	text, Identifier
	305	tmrb_livingordead<br>Show the field ONLY if:<br>[tmrb_yn]=1	403(a). Is (name) living or deceased?	radio<br>1 Living<br>2 Dead
	306	tmrb_fever_yn<br>Show the field ONLY if:<br>[tmrb_livingordead] = '1'	404. Has (Name) been ill with a fever at any time in the last 2 weeks?	radio<br>1 Yes<br>0 No<br>99 Don't Know
	307	tmrb_blooddraw_yn<br>Show the field ONLY if:<br>[tmrb_fever_yn] = '1'	405. At any time during the illness, did (NAME) have blood taken from his/her finger or heel for testing?	radio<br>1 Yes<br>2 No

				3 Don't know
	308	tmrb_adviceortrt_yn<br>Show the field ONLY if:<br>[tmrb_fever_yn] = '1'	406. Did you seek advice or treatment for the illness from any source?	radio<br>1 Yes<br>0 No
	309	tmrb_adviceortrt_whynone<br>Show the field ONLY if:<br>[tmrb_adviceortrt_yn] = '0'	406(a). Why have you not sought advice or treatment from any source?	radio<br>1 Child just fell ill<br>2 Child not very ill<br>3 Clinic too far<br>4 Have no money<br>5 Waiting for child's father<br>6 Don't know what to do<br>7 Already had medicine at home<br>8 Other
	310	tmrb_adviceortrt_whynone_other<br>Show the field ONLY if:<br>[tmrb_adviceortrt_whynone] = '8'	406(a). Specify.	notes
	311	tmrb_adviceortrt_where<br>Show the field ONLY if:<br>[tmrb_adviceortrt_yn] = '1'	407. Where did you seek advice or treatment? Anywhere else?	checkbox<br>1 tmrb_adviceortrt_where___1 Government hospital<br>2 tmrb_adviceortrt_where___2 Government health center<br>3 tmrb_adviceortrt_where___3 Government health post<br>4 tmrb_adviceortrt_where___4 Mobile clinic<br>5 tmrb_adviceortrt_where___5 Community health worker (VHT)<br>6 tmrb_adviceortrt_where___6 Other public sector<br>7 tmrb_adviceortrt_where___7 Public sector (PNFP) hospital<br>8 tmrb_adviceortrt_where___8 Public sector (PNFP) health center<br>9 tmrb_adviceortrt_where___9 Private hospital/clinic<br>10 tmrb_adviceortrt_where___10 Pharmacy<br>11 tmrb_adviceortrt_where___11 Private mobile clinic<br>12 tmrb_adviceortrt_where___12 Fieldworker<br>13 tmrb_adviceortrt_where___13 Other private medical sector<br>14 tmrb_adviceortrt_where___14 Shop<br>15 tmrb_adviceortrt_where___15 Traditional practitioner<br>16 tmrb_adviceortrt_where___16 Market<br>17 tmrb_adviceortrt_where___17 Other

	312	tmrb_adviceortrt_where_other<br>Show the field ONLY if:<br>[tmrb_adviceortrt_yn]='1'	407(a). Specify, and/or if unable to determine if public or private sector, write the name of the place(s).	notes
	313	tmrb_check407<br>Show the field ONLY if:<br>[tmrb_adviceortrt_yn]='1'	408. CHECK 407:	radio<br>1 TWO OR MORE CODES CIRCLED<br>2 ONLY ONE CODE CIRCLED
	314	tmrb_adviceortrt_first_where<br>Show the field ONLY if:<br>[tmrb_check407] = '1'	409. Where did you first seek advice or treatment? USE LETTER CODE FROM 407.<br><br>FIRST PLACE:	text
	315	tmrb_drugstaken_yn<br>Show the field ONLY if:<br>[tmrb_fever_yn] = '1'	410. At any time during the illness, did (NAME) take any drugs for the illness?	radio<br>1 Yes<br>0 No<br>99 Don't know
	316	feverdrug_when	426. I would like to ask you a few questions about fever in children. When a child is sick with fever, how long after the fever begins should the child be taken for treatment?	radio<br>1 Same day<br>2 Next day<br>3 Two days after onset of fever<br>4 Three or more days after onset of fever<br>5 Fever is normal in children, no treatment necessary<br>6 Depends on how serious the fever is<br>7 Other<br>8 Don't know
	317	feverdrug_when_other<br>Show the field ONLY if:<br>[feverdrug_when] = '7'	426(a). Specify.	notes
	318	malaria_cause	427. In your opinion, what causes malaria?	checkbox<br>1 malaria_cause___1 Mosquito bites<br>2 malaria_cause___2 Parasite<br>3 malaria_cause___3 Eating maize<br>4 malaria_cause___4 Eating mangoes<br>5 malaria_cause___5 Eating dirty food<br>6 malaria_cause___6 Drinking unboiled water<br>7 malaria_cause___7 Getting soaked with rain<br>8 malaria_cause___8 Cold/changing weather<br>9 malaria_cause___9 Witchcraft<br>10 malaria_cause___10 Contact with infected person<br>11 malaria_cause___11 Germ<br>12 malaria_cause___12 Other<br>13 malaria_cause___13 Don't know
	319	malaria_cause_other<br>Show the field ONLY if:<br>[malaria_cause(12)] = '1'	427(a). Specify.	notes

	320	malaria_cause_else	427(b). Anything else?	notes
	321	malaria_prevent_yn	428. Are there ways to avoid getting malaria?	yesno<br>1 Yes<br>0 No
	322	malaria_prevent_how<br>Show the field ONLY if:<br>[malaria_prevent_yn] = '1'	429. What are the ways to avoid getting malaria?	checkbox<br>1 malaria_prevent_how___1 Sleep under mosquito net<br>2 malaria_prevent_how___2 Sleep under an insecticide treated net<br>3 malaria_prevent_how___3 Taking preventive medication<br>4 malaria_prevent_how___4 Use mosquito repellant<br>5 malaria_prevent_how___5 Spraying house with insecticide<br>6 malaria_prevent_how___6 Using mosquito coils<br>7 malaria_prevent_how___7 Destroy mosquito breeding sites<br>8 malaria_prevent_how___8 Other<br>9 malaria_prevent_how___9 Don't know
	323	malaria_prevent_how_other<br>Show the field ONLY if:<br>[malaria_prevent_how(8)] = '1'	429(a). Specify.	notes
	324	malaria_prevent_how_else	429(b). Anything else?	notes
	325	malaria_drug_pregnancy	430. What medicine may be given to a pregnant woman to help her avoid getting malaria?	checkbox<br>1 malaria_drug_pregnancy___1 SP/Fansidar<br>2 malaria_drug_pregnancy___2 Chloroquine<br>3 malaria_drug_pregnancy___3 Cholorquine w/Fansidar<br>4 malaria_drug_pregnancy___4 Coartem/Act<br>5 malaria_drug_pregnancy___5 Other<br>6 malaria_drug_pregnancy___6 Don't know
	326	malaria_drug_pregnancy_other<br>Show the field ONLY if:<br>[malaria_drug_pregnancy(5)] = '1'	430(a). Specify.	notes
	327	check_430	431. CHECK 430. SP/FANSIDAR MENTIONED?	radio<br>1 CODE 'A' CIRCLED<br>2 CODE 'A' NOT CIRCLED
	328	malaria_spfansidar_pregnancy_freq<br>Show the field ONLY if:<br>[check_430] = '1'	432. How many times does a woman need to take SP/FANSIDAR during her pregnancy to avoid getting malaria?	radio<br>1 Number of times entered<br>2 Don't know
	329	malaria_spfansidar_pregnancy_times<br>Show the field ONLY if:<br>[malaria_spfansidar_pregnanc	Enter the number of times:	text

		y_freq] = '1'		
	330	illness	Section Header: *Section 5: Respondent Health History*<br>501. Have you ever been told that you have any medical problems, including:	checkbox<br>1 illness___1 Diabetes<br>2 illness___2 Tuberculosis<br>3 illness___3 ISS or HIV<br>4 illness___4 None<br>5 illness___5 Other
	331	illness_other<br>Show the field ONLY if:<br>[illness(5)] = '1'	501(a). Specify.	notes
	332	medication_daily_yn	502. Do you take medications every day?	yesno<br>1 Yes<br>0 No
	333	medication_name<br>Show the field ONLY if:<br>[medication_daily_yn] = '1'	503. What is the name of the medication(s)? (Separate each one with a comma)	notes
	334	hospitalisation_yn	504. Other than for the birth of your children, have you ever had to stay overnight in a hospital or health centre?	yesno<br>1 Yes<br>0 No
	335	hospitalisation_why<br>Show the field ONLY if:<br>[hospitalisation_yn] = '1'	505. What was the reason you had to stay in the hospital or health centre?	radio<br>1 Malaria<br>2 Fever, not malaria<br>3 Diarrhea<br>4 Breathing or pneumonia<br>5 Injury<br>6 Other
	336	hospitalisation_why_other<br>Show the field ONLY if:<br>[hospitalisation_why] = '6'	505(a). Specify.	notes
	337	hiv_consent_yn	506. Prior to beginning the study, we need to confirm your ISS/HIV status as this could impact the study results. This will require a small finger prick. The test will be performed here in your home and we will give you the results. No one else will know.	radio<br>1 Agree<br>2 Not agree
	338	hiv_result<br>Show the field ONLY if:<br>[hiv_consent_yn] = '1'	507. HIV results:	radio<br>1 Negative<br>2 Positive
	339	water_drinking_source	Section Header: *Household Characteristics*<br>101. What is the main source of drinking water for members of your household?	radio<br>1 Piped into dwelling<br>2 Piped to yard/Plot<br>3 Piped to neighbor<br>4 Public tap/Standpipe<br>5 Tube well or borehole<br>6 Protected well<br>7 Unprotected well<br>8 Protected spring

				9 Unprotected spring<br>10 Rainwater<br>11 Tanker truck<br>12 Bicycle with jerrycans<br>13 Surface water (River/Dam/Lake/Pond/Stream/Canal/Irrigation channel)<br>14 Bottled water<br>15 Sachet water<br>16 Other
	340	water_drinking_source_other<br>Show the field ONLY if:<br>[water_drinking_source] = '16'	101(a). Specify.	notes
	341	water_other_source	102. What is the main source of water used by your household for other purposes such as cooking and handwashing?	radio<br>1 Piped into dwelling<br>2 Piped to yard/Plot<br>3 Piped to neighbor<br>4 Public tap/Standpipe<br>5 Tube well or borehole<br>6 Protected well<br>7 Unprotected well<br>8 Protected spring<br>9 Unprotected spring<br>10 Rainwater<br>11 Tanker truck<br>12 Bicycle with jerrycans<br>13 Surface water (River/Dam/Lake/Pond/Stream/Canal/Irrigation channel)<br>14 Other
	342	water_other_source_other<br>Show the field ONLY if:<br>[water_other_source] = '14'	102(a). Specify.	notes
	343	water_source_where	103. Where is that water source located?	radio<br>1 In own dwelling<br>2 In own yard/plot<br>3 Elsewhere
	344	water_time	104. How long does it take to go there, get water, and come back?	radio<br>1 Minutes selected<br>99 Don't know
	345	water_time_min<br>Show the field ONLY if:<br>[water_time] = '1'	Minutes:	text
	346	water_unavailable	106. In the past two weeks, was the water from this source not available for at least one full day?	radio<br>1 Yes<br>0 No

				99 Don't know
	347	water_trt_yn	107. Do you do anything to the water to make it safer to drink?	radio<br>1 Yes<br>0 No<br>99 Don't know
	348	water_trt<br>Show the field ONLY if:<br>[water_trt_yn]='1'	108. What do you usually do to make the water safer to drink?	checkbox<br>1 water_trt___1 Boil<br>2 water_trt___2 Add bleach/chlorine<br>3 water_trt___3 Strain through a cloth<br>4 water_trt___4 Use water filter (Ceramic/Sand/Composite/Etc)<br>5 water_trt___5 Solar disinfection<br>6 water_trt___6 Let it stand and settle<br>7 water_trt___7 Other<br>8 water_trt___8 Don't know
	349	water_trt_other<br>Show the field ONLY if:<br>[water_trt(7)] = '1'	108(a). Specify.	notes
	350	water_trt_else<br>Show the field ONLY if:<br>[water_trt_yn]='1'	108(b). Anything else?	notes
	351	toilet_type	109. What kind of toilet facility do members of your household usually use?	radio<br>1 Flush to piped sewer system<br>2 Flush to septic tank<br>3 Flush to pit latrine<br>4 Flush to somewhere else<br>5 Flush (don't know where)<br>6 Ventilated improved pit latrine<br>7 Pit latrine with slab<br>8 Pit latrine without slab/Open pit<br>9 Composting toilet/Ecosan<br>10 Bucket toilet<br>11 Hanging toilet/Hanging latrine<br>12 No facility/Bush/Field<br>13 Other
	352	toilet_type_other<br>Show the field ONLY if:<br>[toilet_type] = '13'	109(a). Specify.	notes
	353	toilet_share_yn	110. Do you share this toilet facility with other households?	yesno<br>1 Yes<br>0 No
	354	toilet_share_number<br>Show the field ONLY if:<br>[toilet_share_yn] = '1'	111. Including your own household, how many households use this toilet facility?	radio<br>1 'No. of households if less than 10' selected<br>2 10 or more households<br>3 Don't know

	355	toilet_share_numberexact<br>Show the field ONLY if:<br>[toilet_share_number] = '1'	111(a). Number of households:	text
	356	toilet_where	112. Where is this toilet facility located?	radio<br>1 In own dwelling<br>2 In own yard/plot<br>3 Elsewhere
	357	fuel_type	113. What type of fuel does your household mainly use for cooking?	radio<br>1 Electricity<br>2 LPG/Cylinder Gas<br>3 Biogas<br>4 Kerosene<br>5 Charcoal<br>6 Wood<br>7 Straws/Shrubs/Grass<br>8 Agricultural crop<br>9 Animal dung<br>10 No food cooked in household<br>11 Other
	358	fuel_type_other<br>Show the field ONLY if:<br>[fuel_type] = '11'	113(a). Specify.	notes
	359	cooking_where	114. Is the cooking usually done in the house, in a separate building, or outdoors?	radio<br>1 In the house<br>2 In a separate building<br>3 Outdoors<br>4 Other
	360	cooking_where_other<br>Show the field ONLY if:<br>[cooking_where] = '4'	114(a). Specify.	notes
	361	kitchen_separate_yn	115. Do you have a separate room which is used as a kitchen?	yesno<br>1 Yes<br>0 No
	362	rooms_sleeping_number	116. How many rooms in this household are used for sleeping?	text
	363	animals_yn	117. Does this household own any livestock, herds, other farm animals, or poultry?	yesno<br>1 Yes<br>0 No
	364	cattle_local_number<br>Show the field ONLY if:<br>[animals_yn] = '1'	118. How many of the following animals does this household own?<br>a) Local cattle?	text
	365	cattle_exotic_number<br>Show the field ONLY if:<br>[animals_yn] = '1'	b) Exotic/cross-breed cattle?	text
	366	horse_number	c) Horses, donkeys, or mules?	text

		Show the field ONLY if: [animals_yn] ='1'		
	367	goat_number<br>Show the field ONLY if: [animals_yn] ='1'	d) Goats?	text
	368	sheep_number<br>Show the field ONLY if: [animals_yn] ='1'	e) Sheep?	text
	369	chicken_number<br>Show the field ONLY if: [animals_yn] ='1'	f) Chickens or other poultry?	text
	370	pig_number<br>Show the field ONLY if: [animals_yn] ='1'	g) Pigs?	text
	371	animals_inside_yn<br>Show the field ONLY if: [animals_yn] ='1'	118(a). Are there any animals that sleep in the house where people sleep?	yesno<br>1 Yes<br>0 No
	372	land_agriculture_yn	119. Does any member of this household own any agricultural land?	yesno<br>1 Yes<br>0 No
	373	land_agriculture_acres_number<br>Show the field ONLY if: [land_agriculture_yn] = '1'	120. How many acres of agricultural land do members of this household own?	radio<br>1 'Acres' selected<br>2 95 or more acres<br>3 Don't know
	374	land_agriculture_acres_numberexact<br>Show the field ONLY if: [land_agriculture_acres_number] = '1'	How many acres:	text
	375	land_nonagriculture_yn	120(a). Does any member of this household own any non-agricultural land?	yesno<br>1 Yes<br>0 No
	376	electricity_yn	Section Header: *121. Does your household have:*<br>Electricity?	radio (Matrix)<br>1 Yes<br>2 No
	377	radio_yn	A radio?	radio (Matrix)<br>1 Yes<br>2 No
	378	television_yn	A television?	radio (Matrix)<br>1 Yes<br>2 No
	379	nonmobile_yn	A non-mobile telephone?	radio (Matrix)<br>1 Yes<br>2 No
	380	computer_yn	A computer?	radio (Matrix)<br>1 Yes

				2 No
	381	fridge_yn	A refrigerator?	radio (Matrix)<br>1 Yes<br>2 No
	382	musicplayer_yn	A cassette/CD/DVD player?	radio (Matrix)<br>1 Yes<br>2 No
	383	table_yn	A table?	radio (Matrix)<br>1 Yes<br>2 No
	384	chair_yn	A chair?	radio (Matrix)<br>1 Yes<br>2 No
	385	sofa_yn	A sofa set?	radio (Matrix)<br>1 Yes<br>2 No
	386	bed_yn	A bed?	radio (Matrix)<br>1 Yes<br>2 No
	387	cupboard_yn	A cupboard?	radio (Matrix)<br>1 Yes<br>2 No
	388	clock_yn	A clock?	radio (Matrix)<br>1 Yes<br>2 No
	389	watch_yn	Section Header: *122. Does any member of this household own:*<br>A watch?	radio (Matrix)<br>1 Yes<br>2 No
	390	mobilephone_yn	A mobile phone?	radio (Matrix)<br>1 Yes<br>2 No
	391	bike_yn	A bicycle?	radio (Matrix)<br>1 Yes<br>2 No
	392	motorcycle_yn	A motorcycle/scooter?	radio (Matrix)<br>1 Yes<br>2 No
	393	cart_yn	An animal-drawn cart?	radio (Matrix)<br>1 Yes<br>2 No
	394	car_yn	A car/truck?	radio (Matrix)<br>1 Yes<br>2 No

	395	motorboat_yn	A boat with a motor?	radio (Matrix)<br>1 Yes<br>2 No
	396	boat_yn	A boat without a motor?	radio (Matrix)<br>1 Yes<br>2 No
	397	bank_yn	123. Does any member of this household have a bank account, mobile money account, or account with an agent?	yesno<br>1 Yes<br>0 No
	398	smoke_inside_freq	124. How often does anyone smoke inside your house?	radio<br>1 Daily<br>2 Weekly<br>3 Monthly<br>4 Less often than once a month<br>5 Never
	399	spray_walls_yn	125. At any time in the past 6 months, has anyone come into your dwelling to spray the interior walls against mosquitoes?	radio<br>1 Yes<br>0 No<br>2 Don't know
	400	spray_who<br>Show the field ONLY if:<br>[spray_walls_yn] = '1'	126. Who sprayed the dwelling?	radio<br>1 Government worker/program<br>2 Private company<br>3 Nongovernmental organization (NGO)<br>4 Other<br>5 Don't know
	401	spray_who_other<br>Show the field ONLY if:<br>[spray_who] = '4'	(i). Specify.	notes
	402	spray_pay_yn<br>Show the field ONLY if:<br>[spray_walls_yn] ='1'	126(a). Did you pay for your dwelling to be sprayed?	radio<br>1 Yes<br>2 No<br>3 Don't know
	403	net_yn	127. Does your household have any mosquito nets?	yesno<br>1 Yes<br>0 No
	404	net_number<br>Show the field ONLY if:<br>[net_yn] = '1'	128. How many mosquito nets does your household have?<br><br>Number of nets:	text
	405	net_1_seen<br>Show the field ONLY if:<br>[net_number] >= 00	129. Net #1:	radio<br>1 Observed<br>0 Not observed
	406	net_1_when<br>Show the field ONLY if:<br>[net_1_seen] = '1'	130. How many months ago did your household get the mosquito net?	radio<br>1 'Months ago' selected<br>2 More than 36 months ago

				3 Not sure
	407	net_1_months<br>Show the field ONLY if:<br>[net_1_when] = '1'	130(a). Months ago:	text
	408	net_1_brand<br>Show the field ONLY if:<br>[net_1_seen] = '1'	131. Observe or ask brand/type of mosquito net.	radio<br>1 Permanent LLIN<br>2 Duranet LLIN<br>3 Interceptor LLIN<br>4 Olyset LLIN<br>5 Dawnet LLIN<br>6 Iconlife LLIN<br>7 Yorkool LLIN<br>8 DK brand LLIN<br>9 Govt brand LLIN<br>10 Other LLIN<br>11 Other brand<br>12 DK brand
	409	net_1_brand_other<br>Show the field ONLY if:<br>[net_1_brand] = '10'	131(a). Specify.	notes
	410	net_1_trt<br>Show the field ONLY if:<br>[net_1_seen] = 1	132. Since you got the net, was it ever soaked or dipped in a liquid to kill or repel mosquitoes?	radio<br>1 Yes<br>0 No<br>99 Not sure
	411	net_1_trt_when<br>Show the field ONLY if:<br>[net_1_trt] = '1'	133. How many months ago was the net last soaked or dipped?	radio<br>1 'Months ago' selected<br>2 More than 24 months ago<br>99 Not sure
	412	net_1_trt_months<br>Show the field ONLY if:<br>[net_1_trt_when] = '1'	133(a). Months ago:	text
	413	net_1_how<br>Show the field ONLY if:<br>[net_number]>= '1'	134. Did you get the net through a mass distribution, during an antenatal care visit, or during an immunization visit?	radio<br>1 Yes (mass distribution)<br>2 Yes (ANC)<br>3 Yes (immunization visit)<br>4 No
	414	net_1_where<br>Show the field ONLY if:<br>[net_number] >='1'	135. Where did you get the net?	radio<br>1 Govt. hospital<br>2 Govt. health facility<br>3 Hospital (PNFP/NGO)<br>4 Health facility (PNFP/NGO)<br>5 Private hospital/clinic<br>6 Pharmacy<br>7 Shop/market<br>8 Hawker

				9 CHW<br>10 Religious institution<br>11 Other<br>12 Don't know
	415	net_1_sleep_yn<br>Show the field ONLY if:<br>[net_number]>='1'	136. Did anyone sleep under this mosquito net last night?	radio<br>1 Yes<br>0 No<br>99 Not sure
	416	net_1_name_a<br>Show the field ONLY if:<br>[net_1_sleep_yn] = '1'	137. Who slept under this mosquito net last night?<br><br>a) Name:	text
	417	name_b<br>Show the field ONLY if:<br>[net_1_sleep_yn] = '1'	b) Name:	text
	418	net_1_name_c<br>Show the field ONLY if:<br>[net_1_sleep_yn] = '1'	c) Name:	text
	419	net_1_name_d<br>Show the field ONLY if:<br>[net_1_sleep_yn] = '1'	d) Name:	text
	420	net_2_seen<br>Show the field ONLY if:<br>[net_number] >= 2	129. Net #2:	radio<br>1 Observed<br>0 Not observed
	421	net_2_when<br>Show the field ONLY if:<br>[net_2_seen] = '1'	130. How many months ago did your household get the mosquito net?	radio<br>1 'Months ago' selected<br>2 More than 36 months ago<br>99 Not sure
	422	net_2_months<br>Show the field ONLY if:<br>[net_2_when] = '1'	130(a). Months ago:	text
	423	net_2_brand<br>Show the field ONLY if:<br>[net_2_seen] = '1'	131. Observe or ask brand/type of mosquito net.	radio<br>1 Permanent LLIN<br>2 Duranet LLIN<br>3 Interceptor LLIN<br>4 Olyset LLIN<br>5 Dawnet LLIN<br>6 Iconlife LLIN<br>7 Yorkool LLIN<br>8 DK brand LLIN<br>9 Govt brand LLIN<br>10 Other LLIN<br>11 Other brand<br>12 DK brand
	424	net_2_brand_other	131(a). Specify.	notes

		Show the field ONLY if: [net_2_brand] = '10'		
	425	net_2_trt<br>Show the field ONLY if: [net_number] >= 2	132. Since you got the net, was it ever soaked or dipped in a liquid to kill or repel mosquitoes?	radio<br>1 Yes<br>0 No<br>99 Not sure
	426	net_2_trt_when<br>Show the field ONLY if: [net_2_trt] = '1'	133. How many months ago was the net last soaked or dipped?	radio<br>1 'Months ago' selected<br>2 More than 24 months ago<br>99 Not sure
	427	net_2_trt_months<br>Show the field ONLY if: [net_2_trt_when] = '1'	133(a). Months ago:	text
	428	net_2_how<br>Show the field ONLY if: [net_number] >= 2	134. Did you get the net through a mass distribution, during an antenatal care visit, or during an immunization visit?	radio<br>1 Yes (mass distribution)<br>2 Yes (ANC)<br>3 Yes (immunization visit)<br>4 No
	429	net_2_where<br>Show the field ONLY if: [net_number] >= 2	135. Where did you get the net?	radio<br>1 Govt. hospital<br>2 Govt. health facility<br>3 Hospital (PNFP/NGO)<br>4 Health facility (PNFP/NGO)<br>5 Private hospital/clinic<br>6 Pharmacy<br>7 Shop/market<br>8 Hawker<br>9 CHW<br>10 Religious institution<br>11 Other<br>12 Don't know
	430	net_2_sleep_yn<br>Show the field ONLY if: [net_number] >= 2	136. Did anyone sleep under this mosquito net last night?	radio<br>1 Yes<br>2 No<br>3 Not sure
	431	net_2_name_a<br>Show the field ONLY if: [net_2_sleep_yn] = '1'	137. Who slept under this mosquito net last night?<br>a) Name:	text, Identifier
	432	net_2_name_b<br>Show the field ONLY if: [net_2_sleep_yn] = '1'	b) Name:	text, Identifier
	433	net_2_name_c<br>Show the field ONLY if: [net_2_sleep_yn] = '1'	c) Name:	text, Identifier
	434	net_2_name_d	d) Name:	text, Identifier

		Show the field ONLY if: [net_2_sleep_yn] = '1'		
	435	line_no_d_2_8b5ede<br>Show the field ONLY if: [net_2_sleep_yn] = '1'	d) Line number:	text
	436	net_3_seen<br>Show the field ONLY if: [net_number] >= 3	129. Net #3:	radio<br>1 Observed<br>0 Not observed
	437	net_3_when<br>Show the field ONLY if: [net_3_seen] = '1'	130. How many months ago did your household get the mosquito net?	radio<br>1 'Months ago' selected<br>2 More than 36 months ago<br>3 Not sure
	438	net_3_months<br>Show the field ONLY if: [net_3_when] = '1'	130(a). Months ago:	text
	439	net_3_brand<br>Show the field ONLY if: [net_3_seen] = '1'	131. Observe or ask brand/type of mosquito net.	radio<br>1 Permanent LLIN<br>2 Duranet LLIN<br>3 Interceptor LLIN<br>4 Olyset LLIN<br>5 Dawnet LLIN<br>6 Iconlife LLIN<br>7 Yorkool LLIN<br>8 DK brand LLIN<br>9 Govt brand LLIN<br>10 Other LLIN<br>11 Other brand<br>12 DK brand
	440	net_3_brand_other<br>Show the field ONLY if: [net_3_brand] = '10'	131(a). Specify.	notes
	441	net_3_trt<br>Show the field ONLY if: [net_number] >= 3	132. Since you got the net, was it ever soaked or dipped in a liquid to kill or repel mosquitoes?	radio<br>1 Yes<br>0 No<br>99 Not sure
	442	net_3_trt_when<br>Show the field ONLY if: [net_3_trt] = '1'	133. How many months ago was the net last soaked or dipped?	radio<br>1 'Months ago' selected<br>2 More than 24 months ago<br>99 Not sure
	443	net_3_trt_months<br>Show the field ONLY if: [net_3_trt_when] = '1'	133(a). Months ago:	text
	444	net_3_how<br>Show the field ONLY if: [net_number] >= 3	134. Did you get the net through a mass distribution, during an antenatal care visit, or during an immunization visit?	radio<br>1 Yes (mass distribution)<br>2 Yes (ANC)<br>3 Yes (immunization visit)

				4 No
	445	net_3_where<br><br>Show the field ONLY if:<br>[net_number] >= 3	135. Where did you get the net?	radio<br>1 Govt. hospital<br>2 Govt. health facility<br>3 Hospital (PNFP/NGO)<br>4 Health facility (PNFP/NGO)<br>5 Private hospital/clinic<br>6 Pharmacy<br>7 Shop/market<br>8 Hawker<br>9 CHW<br>10 Religious institution<br>11 Other<br>12 Don't know
	446	net_3_sleep_yn<br><br>Show the field ONLY if:<br>[net_number] >= 3	136. Did anyone sleep under this mosquito net last night?	radio<br>1 Yes<br>0 No<br>99 Not sure
	447	net_3_name_a<br><br>Show the field ONLY if:<br>[net_3_sleep_yn] = '1'	137. Who slept under this mosquito net last night?<br><br>a) Name:	text, Identifier
	448	net_3_name_b<br><br>Show the field ONLY if:<br>[net_3_sleep_yn] = '1'	b) Name:	text, Identifier
	449	net_3_name_c<br><br>Show the field ONLY if:<br>[net_3_sleep_yn] = '1'	c) Name:	text, Identifier
	450	net_3_name_d<br><br>Show the field ONLY if:<br>[net_3_sleep_yn] = '1'	d) Name:	text, Identifier
	451	handwash_where	Section Header: *Additional Household Characteristics*<br>139. We would like to learn about the places that households use to wash their hands. Can you please show me where members of your household most often wash their hands?	checkbox<br>1 handwash_where___1 Observed (fixed place)<br>2 handwash_where___2 Observed (mobile)<br>3 handwash_where___3 Not observed (not in dwelling/yard/plot)<br>4 handwash_where___4 Not observed (no permission to see)<br>5 handwash_where___5 Not observed (other reason)
	452	handwash_water_yn<br><br>Show the field ONLY if:<br>[handwash_where(2)] = '1' or [handwash_where(1)] = '1'	140. Observe presence of water at the place for handwashing.<br><br>Record observation.	radio<br>1 Water is available<br>0 Water is not available
	453	handwash_soap_type<br><br>Show the field ONLY if:<br>[handwash_where(1)] = '1' or [handwash_where(2)] = '1'	141. Observe presence of soap, detergent, or other cleansing agent at the place for handwashing.<br><br>Record observation.	checkbox<br>1 handwash_soap_type___1 Soap or detergent (bar/liquid/powder/paste)

			2 handwash_soap_type___2 Ash/mud/sand<br>3 handwash_soap_type___3 None
454	floor_material	142. Observe main material of the floor of the dwelling.<br><br>Record observation.	checkbox<br>1 floor_material___1 Earth/sand<br>2 floor_material___2 Dung<br>3 floor_material___3 Wood planks<br>4 floor_material___4 Palm/Bamboo<br>5 floor_material___5 Parquet or polished wood<br>6 floor_material___6 Concrete<br>7 floor_material___7 Ceramic tiles<br>8 floor_material___8 Cement screed<br>9 floor_material___9 Carpet<br>10 floor_material___10 Stones<br>11 floor_material___11 Bricks<br>12 floor_material___12 Other
455	floor_material_other<br>Show the field ONLY if:<br>[floor_material(12)] = '1'	142(a). Specify.	notes
456	roof_material	143. Observe main material of the roof of the dwelling.<br><br>Record observation.	checkbox<br>1 roof_material___1 No roof<br>2 roof_material___2 Thatch/palm leaf<br>3 roof_material___3 Mud<br>4 roof_material___4 Rustic mat<br>5 roof_material___5 Tins<br>6 roof_material___6 Wood planks<br>7 roof_material___7 Cardboard<br>8 roof_material___8 Tarpaulin<br>9 roof_material___9 Iron sheets<br>10 roof_material___10 Wood<br>11 roof_material___11 Asbestos<br>12 roof_material___12 Tiles<br>13 roof_material___13 Concrete<br>14 roof_material___14 Roofing Shingles<br>15 roof_material___15 Other
457	roof_material_other<br>Show the field ONLY if:<br>[roof_material(15)] = '1'	143(a). Specify.	notes
458	exteriorwalls_material	144. Observe main material of the exterior walls of the dwelling. Record observation.	checkbox<br>1 exteriorwalls_material___1 No walls<br>2 exteriorwalls_material___2 Thatched/straw<br>3 exteriorwalls_material___3 Dirt<br>4 exteriorwalls_material___4 Poles with mud<br>5 exteriorwalls_material___5 Stone with mud<br>6 exteriorwalls_material___6 Unburnt bricks with mud

				7 exteriorwalls_material___7 Plywood<br>8 exteriorwalls_material___8 Cardboard<br>9 exteriorwalls_material___9 Reused wood<br>10 exteriorwalls_material___10 Unburnt bricks with plaster<br>11 exteriorwalls_material___11 Burnt bricks with mud<br>12 exteriorwalls_material___12 Cement<br>13 exteriorwalls_material___13 Stone with lime/cement<br>14 exteriorwalls_material___14 Burnt bricks with cement<br>15 exteriorwalls_material___15 Cement blocks<br>16 exteriorwalls_material___16 Unburnt bricks with cement<br>17 exteriorwalls_material___17 Wood planks/shingles<br>18 exteriorwalls_material___18 Other
	459	exteriorwalls_material_other<br>Show the field ONLY if:<br>[exteriorwalls_material(18)] = '1'	144(i). Specify.	notes
	460	healthcare_where	144(a). Where do you and your family mainly go for health care?	radio<br>1 Government hospital<br>2 Government health center<br>3 Family planning clinic<br>4 Mobile clinic<br>5 Other public sector<br>6 Private hospital/clinic<br>7 Private doctor<br>8 Mobile private clinic<br>9 Pharmacy/drug shop<br>10 Other private medical center<br>11 Other<br>12 Don't know
	461	healthcare_where_other	ii. Specify. If unable to determine if public or private sector, write the name of the place.	notes
	462	healthcare_pay_yn	144(b). Do you pay any money for the services offered?	radio<br>1 Yes (official fees)<br>2 Yes (token of thanks)<br>3 No<br>4 Don't know
	463	healthcare_pay_how<br>Show the field ONLY if:<br>[healthcare_pay_yn] = '1' or [healthcare_pay_yn] = '2'	144(c). How do you make the payment?	radio<br>1 Directly out of pocket<br>2 Community-based initiative/savings<br>3 Health insurance through employer<br>4 Social security

				5 Other privately purchased commercial health insurance<br>6 Other
	464	healthcare_pay_how_else<br><br>Show the field ONLY if:<br>[healthcare_pay_how] = '6'	(i). Specify.	notes
	465	home_visit_d08c3f_complete	Section Header: *Form Status*<br><br>Complete?	dropdown<br>0 Incomplete<br>1 Unverified<br>2 Complete
